# Verapamil inhibits efflux pumps in *Candida albicans*, exhibits synergism with fluconazole, and increases survival of *Galleria mellonella*

**DOI:** 10.1080/21505594.2020.1868814

**Published:** 2021-01-07

**Authors:** Yuliana Vega-Chacón, Maria Carolina de Albuquerque, Ana Cláudia Pavarina, Gustavo Henrique Goldman, Ewerton Garcia de Oliveira Mima

**Affiliations:** a Laboratory of Applied Microbiology, Department of Dental Materials and Prosthodontics, São Paulo State University (UNESP), School of Dentistry, Araraquara, Brazil; b Faculdade de Ciências Farmacêuticas de Ribeirão Preto, Universidade de São Paulo (USP), Ribeirão Preto, Brazil

**Keywords:** *Candida albicans*, membrane transport proteins, virulence, drug resistance, *Galleria mellonella*

## Abstract

The emergence of resistance requires alternative methods to treat *Candida albicans* infections. We evaluated efficacy of the efflux pump inhibitor (EPI) verapamil (VER) with fluconazole (FLC) against FLC-resistant (CaR) and -susceptible *C. albicans* (CaS). The susceptibility of both strains to VER and FLC was determined, as well as the synergism of VER with FLC. Experiments were performed *in vitro* for planktonic cultures and biofilms and *in vivo* using *Galleria mellonella*. Larval survival and fungal recovery were evaluated after treatment with VER and FLC. Data were analyzed by analysis of variance and Kaplan-Meier tests. The combination of VER with FLC at sub-lethal concentrations reduced fungal growth. VER inhibited the efflux of rhodamine 123 and showed synergism with FLC against CaR. For biofilms, FLC and VER alone reduced fungal viability. The combination of VER with FLC at sub-lethal concentrations also reduced biofilm viability. In the *in vivo* assays, VER and FLC used alone or in combination increased the survival of larvae infected with CaR. Reduction of fungal recovery was observed only for larvae infected with CaR and treated with VER with FLC. VER reverted the FLC-resistance of *C. albicans*. Based on the results obtained, VER reverted the FLC-resistance of *C. albicans* and showed synergism with FLC against CaR. VER also increased the survival of *G. mellonella* infected with CaR and reduced the fungal recovery.

## Introduction

Oral candidiasis is the most common fungal infection of the oral cavity, and its main etiological agent is *Candida albicans* [1,2]. In immunosuppressed patients, this infection can spread to the bloodstream causing candidemia, which is one of the main nosocomial infections associated with high mortality rates ranging from 25%-60% [3].

Microbial infections, including oral candidiasis, are strongly associated with biofilms, which are communities of microorganisms attached to a biotic or abiotic surface that are embedded in an extracellular polymeric matrix [4–6]. Compared to their free-floating (planktonic) counterparts, cells growing as part of biofilms exhibit distinct phenotypic properties and have a greater tolerance toward antimicrobial agents [7,8].

The misuse and overuse of conventional antifungal agents has raised the problem of antifungal resistance [9]. According to the World Health Organization, antimicrobial resistance threatens public health and is a global concern [10]. Persistent infections caused by resistant strains are difficult to treat and costly due to long hospital stays. Some resistance mechanisms of *C. albicans* have been identified, especially against azole drugs. These mechanisms include genetic mutations and chromosomal aberrations [7], overexpression of plasma membrane multidrug transporters (efflux pumps, EPs), and signaling via cellular stress response pathways [7,9,11].

EPs or microbial efflux systems are membrane proteins that transport toxic substances out of the cell and have been widely recognized as the main mediators of microbial resistance toward several classes of antimicrobial drugs [9,12]. In *C. albicans* there are two important classes of efflux systems that are responsible for drug resistance: the energy-dependent transporter classes, or ATP-binding cassette (ABC) transporter superfamily, and the major facilitator superfamily (MFS) [13–16]. The ABC transporters *Cdr1p* and *Cdr2p*, and the MFS transporter *Mdr1p* are responsible for azole resistance [13–18]. The EPs can export a wide range of structurally unrelated compounds, such as antifungal drugs, herbicides, steroids, lipids, fluorescent dyes, etc [19]. Thus, the inhibition of EPs is considered as an important method for combating microbial resistance [20].

Several approaches have been proposed to address the antimicrobial drug resistance mediated by EP, such as the direct pharmacological inhibition of efflux systems [21–23]. Studies have shown that certain drugs can be used to inhibit EPs that are localized at the fungal plasma membrane, such as verapamil (VER) [24–26]. VER is a calcium channel blocker of the phenylalkylamine class and is used to treat hypertension [27] and angina pectoris [28]. In *C. albicans*, VER inhibits the metabolic activity of biofilms, shows synergism with fluconazole (FLC) [24], inhibits fungal filamentation [26], and reduces the expression of genes responsible for cellular adhesion and the oxidative stress response [24–26]. Therefore, VER is a promising efflux pump inhibitor (EPI) and its combination with FLC can increase *in vitro* the susceptibility of FLC-resistant *C. albicans* to antifungal inactivation [24]. However, the *in vivo* effect of VER on FLC-resistant *C. albicans* is not known. In this study, we investigated the inhibition of EPs for the reversion of FLC-resistance in *C. albicans in vitro* and *in vivo* using the greater wax moth *Galleria mellonella*.

## Materials and methods

Initially, we investigated the use of curcumin (CUR) and VER as EPIs against FLC-resistant *C. albicans* (CaR). CUR was also used as a photosensitizer (PS) for antimicrobial photodynamic therapy against CaR, as other PSs such as methylene blue are substrates for EPs [21,22,29]. Because VER showed better results than CUR as an EPI, the results obtained with CUR are described in the Supplemental Material.

### Preparation of drugs

FLC (Sigma-Aldrich, St. Louis, MO) was added to Yeast Nitrogen Broth (YNB; Difco, Detroit, MI, USA) with 2.5% DMSO, which was not toxic toward *C. albicans* (Figure S1). VER hydrochloride (Sigma-Aldrich, St. Louis, MO) was used as an inhibitor of the fungal efflux system and was dissolved in sterile ultra-pure water immediately before using.

### Candida albicans *strains and growth conditions*

An FLC-susceptible (CaS; ATCC®90028™, American Type Culture Collection, Rockville, MD, USA) and an FLC-resistant standard *C. albicans* strain (CaR, ATCC 96901) were evaluated. The strains were stored at −80°C in YNB with 50% glycerol. Each strain was individually thawed and plated onto Sabouraud Dextrose Agar (SDA; Acumedia Manufacturers Inc., Lansing, MI, USA) culture medium having 0.05 mg/mL chloramphenicol. After incubation at 37°C for 48 h, five colonies were transferred to YNB medium having 100 mM glucose (YNBg) and incubated at 37°C overnight. Next, each fungal suspension was diluted 1:20 in fresh YNB medium and were incubated at 37°C until an optical density at 540 nm (OD_540_; Bioespectro SP 220 Equipar Ltda, Curitiba, PR, Brazil) was reached such that the cells were in the mid-log phase of growth before the planktonic culture and biofilm assays were performed. At this growth point, the mean value ± standard deviation (SD) at OD_540_ was 0.658 ± 0.091 and 0.514 ± 0.123 arbitrary units (a.u.) for the CaS and CaR strains, respectively, which corresponded to a mean ± SD value of 4.14 × 10^6^ ± 2.29 × 10^5^ and 3.61 × 10^6^ ± 9.16 × 10^5^ colony forming units per milliliter (CFU/mL), respectively.

### Planktonic cultures

#### Susceptibility test

The minimum inhibitory concentration (MIC) and minimum fungicidal concentration of each agent (VER and FLC) were evaluated by the microdilution method, based on the recommendations of the Clinical and Laboratory Standards Institute (CLSI, M27-A3) [30] and the European Committee for Antimicrobial Susceptibility Testing (EUCAST) [31], with some modifications. Briefly, 100 μL of each drug (VER and FLC) was serially diluted two-fold in YNBg in 96-well, U-bottom microtiter plates (TPP Techno Plastic Products, Trasadingen, Switzerland). The final concentrations of the drugs used for both strains are shown in Table 1. Next, the fungal suspensions were diluted to 10^3^ CFU/mL, and 100 μL of each strain was added to each well at a final concentration of 0.5–2.5 × 10^3^ CFU/mL. Fungal suspensions with drug vehicles (2.5% DMSO) alone were used as no-drug controls, drugs in YNBg without fungal suspension were used as controls for sterile conditions, and YNBg alone was used as blank. The plates were incubated at 37°C for 24 h, and the OD_540_ was determined before and after incubation in a spectrophotometer (FLUOstar Omega microplate reader; BMG Labtech GmbH, Offenburg, Germany). The MIC was identified as the lowest concentration of drug that caused no increase in the OD value after incubation. Additionally, for samples with OD values similar or higher than the MIC, the contents of each well (100 μL) were serially diluted and plated onto SDA, and were incubated at 37°C for 48 h to estimate colony viability (CFU/mL).

**Table 1.** Drug concentrations for the CaS and CaR strains used in the susceptibility test

Drugs	CaS	CaR
VER (mg/mL)	2, 4, 8, 16	2, 4, 8, 16
FLC (μg/mL)	0.031, 0.062, 0.125, 0.25, 0.5, 1, 2, 4	8, 16, 32, 64, 128, 256, 512, 1024

VER: verapamil; FLC: fluconazole; CaS: FLC-susceptible *C. albicans*; CaR: FLC-resistant *C. albicans*.

#### Efflux of rhodamine 123 (Rh123)

To evaluate the EP action, a rhodamine incorporation test was performed. Planktonic cultures of CaS and CaR strains (4.09 × 10^6^ ± 2.12x10^5^ CFU/mL) were incubated at 37°C with 10 µM Rh123 (Sigma-Aldrich®, St Louis, MO, USA) with 2% glucose and 0.5 g/L calcofluor white (Sigma-Aldrich) in 10% KOH for 2 h. The samples were centrifuged at 5,000 x*g* for 5 min at 4°C and washed twice with phosphate-buffered saline (PBS; 0.136 M NaCl; 1 mM KH_2_PO_4_; 2 mM KCl; 10 mM Na_2_HPO_4_; pH 7.4). A solution of 2 mg/mL VER was prepared using PBS and 2% glucose. Fungal samples were incubated with VER for 1 h at 37°C. After the incubation, a final concentration of 10 μM Rh123 was added and the cells were incubated for 2 h at 37°C, centrifuged (5,000 x*g*, 5 min, 4°C), and washed with PBS. Microscope slides of the cells were prepared and observed using a laser scanning confocal microscope (Carl Zeiss LSM 800 with Airyscan, Germany) at excitation/emission wavelengths of 485/538 nm and 405/470 nm for Rh123 and calcofluor white, respectively.

#### Inhibition of fungal efflux systems

Non-lethal concentrations (sub-MIC) of VER (2 mg/mL for CaS and CaR) were combined with FLC (0.25 μg/mL for CaS, and 64 μg/mL for CaR) for each strain as described above to evaluate the inhibition of the efflux system. Briefly, 50 μL of VER was added to 50 μL of FLC in the wells of a microtiter plate, and the fungal suspension was added at a final concentration of 0.5–2.5 × 10^3^ CFU/mL. The drug combination was prepared so that the final concentration of each drug in the fungal inoculum was at sub-MIC levels. The samples were incubated at 37°C for 24 h, the OD_540_ was determined, plating was done on SDA, and the medium was incubated at 37°C for 48 h. The controls used were fungal suspensions in YNB with drug vehicles alone and blank medium with drugs but without fungal suspensions.

#### Interaction of EPIs with FLC

The checkerboard microdilution assay was performed to evaluate the interaction of VER with FLC following the standards of the CLSI [30] and the EUCAST [31] with some modifications. Two-fold serial dilutions of FLC (50 µL) and VER (50 µL) were distributed along the rows and columns, respectively, of a 96-well, U-bottom microtiter plate (Kasvi, São José dos Pinhais, Brazil). The final drug concentrations used for each strain are shown in Table 2. An aliquot of 100 µL of CaS or CaR was individually added at final concentrations of 0.5–2.5 × 10^3^ CFU/mL. The control consisted of fungal inoculum without drug (vehicle only). After 24 h of incubation at 37°C, the OD_540_ was determined, the control and the samples with a lower OD value than the control were diluted and plated on SDA for colony counting.

**Table 2.** Concentrations of drugs used in the Checkerboard assay

Drugs	CaS	CaR
VER (mg/mL)	0, 0.5, 1, 2, 4	0, 0.5, 1, 2, 4
FLC (µg/mL)	0, 0.032, 0.063, 0.125, 0.25, 0.5	0, 0.5, 1, 2, 4, 8, 16, 32, 64, 128

VER: verapamil; FLC: fluconazole; CaS: FLC-susceptible *C. albicans*; CaR: FLC-resistant *C. albicans*.

To assess the interaction between the drugs, the fractional inhibitory concentration index (FICI) [32] was determined using the sum of the FICI of each agent (FICI = FICI_VER_ + FICI_FLC_). The FICI of each agent was calculated by dividing the MIC of the agent in combination with the MIC of the agent alone (FICI_A_ = MIC_A in the presence of B_/MIC_A alone_). The FICI value was interpreted as follows: FICI < 0.5: synergism; 0.5 ≤ FICI ≤ 4.0: no interaction; and FICI > 4.0: antagonism [33]. In addition, the Bliss independence model [34–36] was used due to the deficiencies of the FICI method [34]. The Bliss model is based on the idea that each drug acts independently of each other, and is calculated by the following equation: E_IND_ = E_A_ + E_B_ – E_A_ × E_B_, for a combination of drug A at concentration *a*, and drug B at concentration *b*. E_A_ and E_B_ are the percentages of growth inhibition observed for drug A or B alone at concentration *a* or *b*, respectively, and E_IND_ is the expected percentage of growth inhibition of a non-interactive combination of drug A at *a* with drug B at *b*. The difference (ΔE = E_OBS_ – E_IND_) between the observed growth inhibition percentage (E_OBS_) and the expected percentage (E_IND_) describes the drug interaction for each concentration as follows: when ΔE and its 95% confidence interval (CI) were > 0, synergism was concluded. If ΔE and the 95% CI were < 0, antagonism was concluded for that combination, and Bliss independence was concluded when the 95% CI of ΔE overlapped 0 [35,36]. Experiments were performed thrice, and the FICI and Bliss independence analyses were performed for each drug combination using OD_540_ values. The mean ΔE values were used to build a three-dimensional surface graph, where the peaks above the plane 0 corresponded to synergism, valleys below 0 corresponded to antagonism, and the plane 0 indicated no statistically significant interaction.

### Biofilms

For biofilm formation, 200 μL of the standardized fungal suspensions (4.17 x 10^6^ ± 6.99 x 10^5^ CFU/mL) were transferred to 96-well, flat-bottom microtiter plates (Kasvi) and incubated at 37°C for 90 min (adhesion phase) with agitation at 75 rpm. After incubation, the wells were washed with 200 μL of sterile PBS twice to remove non-adherent cells. Next, 200 μL of bicarbonate free Roswell Park Memorial Institute 1640 medium (RPMI; Sigma-Aldrich), buffered with morpholinepropanesulfonic acid (MOPS; Sigma-Aldrich), and supplemented with 2% D-glucose (Synth, São Paulo, Brazil), pH 7.0 (RPMIg) was added to the wells, and the plates were incubated for 48 h at 37°C for biofilm formation. After an initial 24 h of incubation, 100 μL of the content from each well was removed and renewed by adding 100 μL of fresh RPMIg, and the plates were incubated for a further 24 h [37].

#### Susceptibility testing

After biofilm formation, samples were washed twice with PBS and 200 μL of the drugs were added. The final concentrations of drugs for both strains are shown in Table 3. Control biofilms were not treated with any drug and received the same volume of drug vehicle. All samples were incubated at 37°C for 24 h. After incubation, biofilms were washed twice with PBS and mechanically disrupted using a pipette tip and 200 μL of PBS for serial dilutions, which were plated on SDA and incubated at 37°C for 48 h, and the resulting colonies were counted.

**Table 3.** Drug concentrations for the biofilms of CaS and CaR strains used in the susceptibility test

Drugs	CaS	CaR
VER (mg/mL) FLC (μg/mL)	4, 8, 16 0.5, 1, 2, 4, 8	4, 8, 16 32, 64, 128, 256, 512, 1024

VER: verapamil; FLC: fluconazole; CaS: FLC-susceptible *C. albicans*; CaR: FLC-resistant *C. albicans*.

#### Inhibition of fungal efflux systems

The highest non-lethal concentration of VER was combined with the highest non-lethal concentration of FLC to verify the potential reversal of FLC-resistance. The concentrations of VER and FLC used were 4 mg/mL and 1 μg/mL, respectively, for CaS, and 4 mg/mL and 64 μg/mL, respectively, for CaR. The biofilms were washed, a final volume of 200 μL of the combined drugs VER + FLC was added, and the mix was incubated at 37°C for 24 h. The control samples received the drug vehicle. After the incubation, the biofilms were washed twice with PBS, disrupted, and plated on SDA for colony counting as described above.

### In vivo *assays*

#### *FLC and VER on the survival of* G. mellonella *infected with* C. albicans

Larvae in the final stage of development (sixth instar) of average size (approximately 150 to 200 mg) were selected. Different 10 µL Hamilton microsyringes (Fisher Scientific, Buenos Aires, Argentina) were used to inject the fungal suspensions and drugs into the larvae, which were previously cleaned for 10 min using 10% bleach, then with 100% ethanol, distilled water, and finally with sterile PBS [38]. FLC was prepared with 2.5% DMSO and sterile saline. VER was diluted using sterile saline. Suspensions of CaR and CaS were centrifuged (6,000 ×*g*, 10 min, 4°C), washed twice, and resuspended in sterile saline. The mean OD_540_ values for CaS and CaR were 0.655 ± 0.098 a.u. and 0.560 ± 0.140 a.u., which corresponded to a mean ± SD value of 1.57 × 10^7^ ± 6.99 × 10^6^ CFU/mL and 1.49 × 10^7^ ± 2.40 × 10^6^ CFU/mL, respectively.

For fungal inoculation, each larva was handled with light pressure and 10 μL of CaS or CaR was injected at the last, left pro-leg [38,39]. The larvae were incubated at 33°C, and after 2 h, 10 μL of the drugs (VER and FLC) were injected, alone or in combination, in the last, right pro-leg. The following groups were evaluated (n = 10 each): control (fungal inoculum and saline); FLC (fungal inoculum and FLC); VER (fungal inoculum and VER); VER + FLC (fungal inoculum and VER combined with FLC). Each drug was used at its MIC. In another group (saline), larvae were injected with sterile saline in both the right and left pro-legs to assess the effect of the injection trauma. After the injections, the larvae were kept in separate Petri dishes according to each group, incubated at 33°C, and were observed daily for survival until no larvae were left or they became pupae. To assess larval survival, they were lightly touched to verify the lack of response to the stimulus [38–40].

#### Fungal recovery from G. mellonella

The fungal load was determined at 5 days after infection. Larvae were classified into the same groups (n = 10) as described above and, every 24 h, two larvae from each group were selected. Each larva was homogenized in 1 mL of sterile saline [40], and serial dilutions were plated on SDA. The plates were incubated at 37°C for 48 h for colony counting.

### Statistical analysis

Each *in vitro* experiment was performed in quadruplicate thrice or five times (n = 3 or 5 for each group). The data [log_10_ (CFU/mL)] were analyzed using the Shapiro-Wilk and Levene tests to verify the normal distribution and homogeneity of variances, respectively. The data were analyzed using two-way ANOVA (with strain and treatment as independent variables). For homoscedastic data, the *post-hoc* Tukey’s test was used. When data were heteroscedastic, they were evaluated using the *post-hoc* Games–Howell test. The survival curves of *G. mellonella* were analyzed via the Kaplan-Meier method and log-rank tests, and the fungal loads were analyzed using three-way ANOVA (strain, treatment, and recovery day as independent factors). The level of significance was 5%, and the SPSS software (version 25.0, SPSS Inc., Chicago, IL, USA) was used for all statistical analyses.

## Results

### Planktonic cultures

#### Susceptibility test

The MIC values of VER and FLC were estimated for the CaS and CaR strains, and the reductions in the log_10_ (CFU/mL) values are shown in Table 4.

**Table 4.** Minimum inhibitory concentrations of FLC and VER against both strains of *C. albicans.*

Strain	FLC	VER
CaS	0.5 μg/mL (2.12 log_10_)	4 mg/mL (4.05 log_10_)
CaR	128 μg/mL (2.27 log_10_)	4 mg/mL (1.45 log_10_)

The values in parentheses show the reduction in viability compared with the control (without drug). VER: verapamil; FLC: fluconazole; CaS: FLC-susceptible *C. albicans*; CaR: FLC-resistant *C. albicans*.

#### Efflux of Rh123

**Figure 1.**
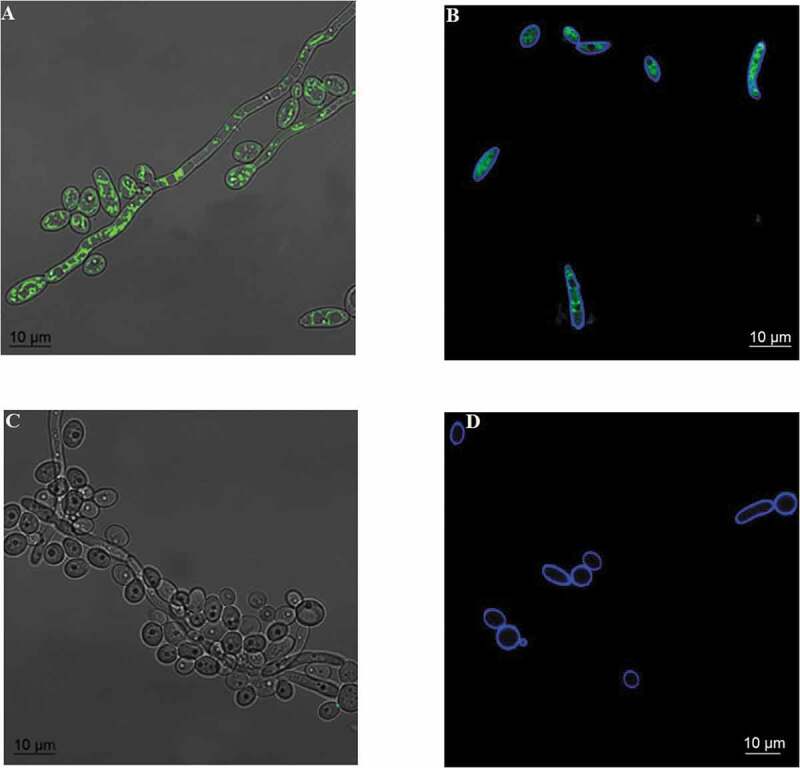
Incorporation of rhodamine (green) and cell wall labeling with calcofluor white (blue). The images show the overlapping of bright-field and rhodamine fluorescence in the CaS (A) and CaR (C) strains and overlapping fluorescence images of rhodamine and calcofluor white in the CaS (B) and CaR (D) strains

The CaS cells showed intracellular retention of Rh123 (green fluorescence), whereas the CaR cells did not exhibit Rh123 fluorescence, suggesting that Rh123 was a substrate for the EPs of CaR strains (Figure 1).

**Figure 2.**
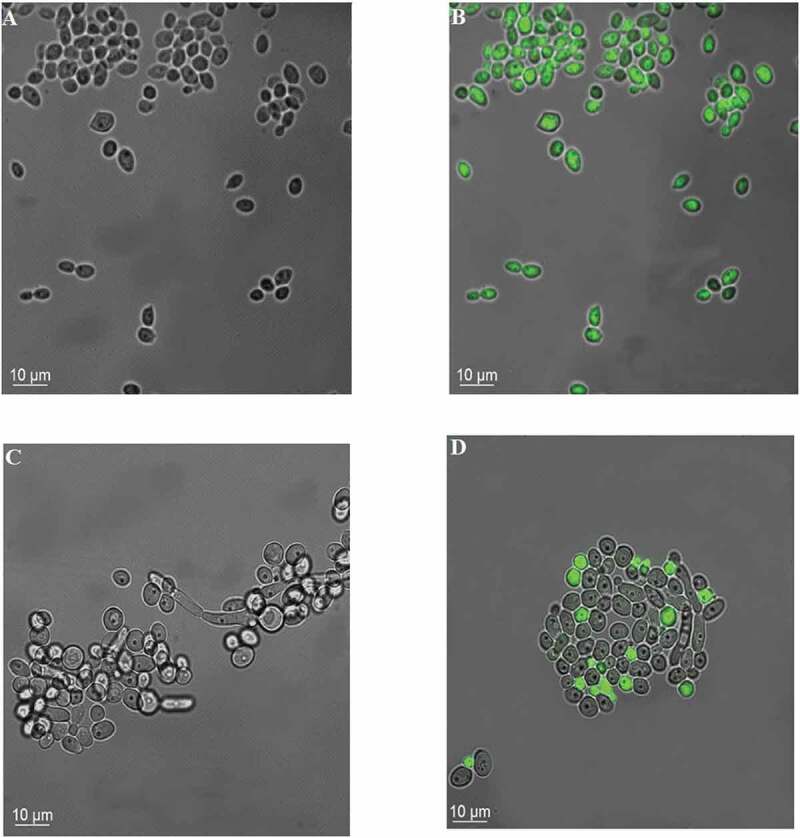
Incorporation of rhodamine (green) after the treatment of fungal suspensions with VER. The images show bright-field images of CaS (A) and CaR (C) strains, and the overlapping of the bright-field and rhodamine fluorescence images of CaS (B) and CaR (D) strains

CaR cells treated with VER at 2 mg/mL showed the intracellular retention of Rh123 (green fluorescence), suggesting that VER prevented Rh123 from being a substrate of the EPs in the CaR strain (Figure 2).

#### Inhibition of fungal efflux systems

After establishing the MICs and the concentrations of drugs that inhibited the growth of both *C. albicans* strains, VER was combined with FLC at sub-MIC values, i.e., 2 mg/mL VER was used for both strains in combination with 0.25 and 64 μg/mL FLC for CaS and CaR, respectively.

**Figure 3.**
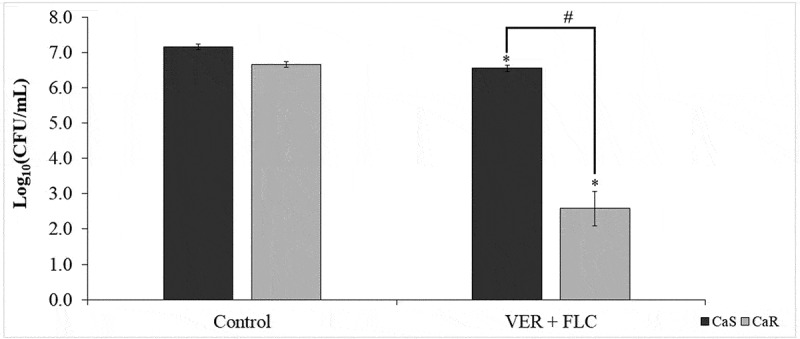
Mean values of log_10_ (CFU/mL) were calculated for both strains after 24 h of incubation with 2 mg/mL VER combined with 0.25 μg/mL (CaS) or 64 μg/mL (CaR) of FLC. Error bars: standard deviation (n = 3). (*) indicates the significant difference between the treated and control groups for the same strain, and (**#**) indicates the significant difference between the strains for the same treatment (*p* < 0.05)

The two-way ANOVA indicated a significant interaction (*p* < 0.001) between the

strains and the treatment with VER and FLC. The combination of VER and FLC resulted in a greater growth reduction for CaR (4.08 log_10_, *p* < 0.001) than that observed for CaS (0.60 log_10_, *p* < 0.001) compared with their respective controls (Figure 3).

#### Interaction of EPIs with FLC

The checkerboard assay with VER and FLC for CaS showed no interaction between the drugs (FICI values ranging from 0.625 to 1.000) and high mean values for CFU/mL. For CaR, the FICI values ranged from 0.508 to 1.000, which corresponded to no interaction between VER and FLC, and the plated samples showed mean values ranging from 4.47 × 10^3^ to 4.92 × 10^6^ CFU/mL.

**Figure 4.**
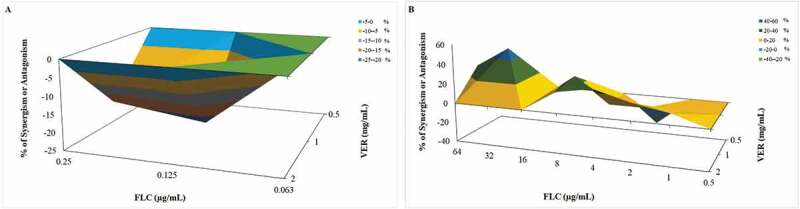
Three-dimensional surface graph representing the Bliss independence analyses of the interaction between VER and FLC for CaS (A) and CaR (B). The concentrations of FLC and VER are shown in X and Z-axes, respectively, and the Y-axis shows the % value of ΔE. Peaks above the 0 plane represents synergism (%), valleys below the 0 plane represent antagonism (%), and the 0 plane represents no interaction (95% CI overlapped at 0)

The Bliss independence analysis showed antagonism between 1 mg/mL VER and 0.25 and 0.125 µg/mL FLC for CaS (Figure 4a; CI of −0.096 – −0.280 and −0.073 – −0.378, respectively), but showed synergism between VER at 2 and 1 mg/mL and FLC at 64, 32, 8, 4, 2, and 1 µg/mL for the CaR strain (Figure 4b; CI from 0.166–0.055 and 0.943–0.061, respectively).

### Biofilms

#### Susceptibility test

**Figure 5.**
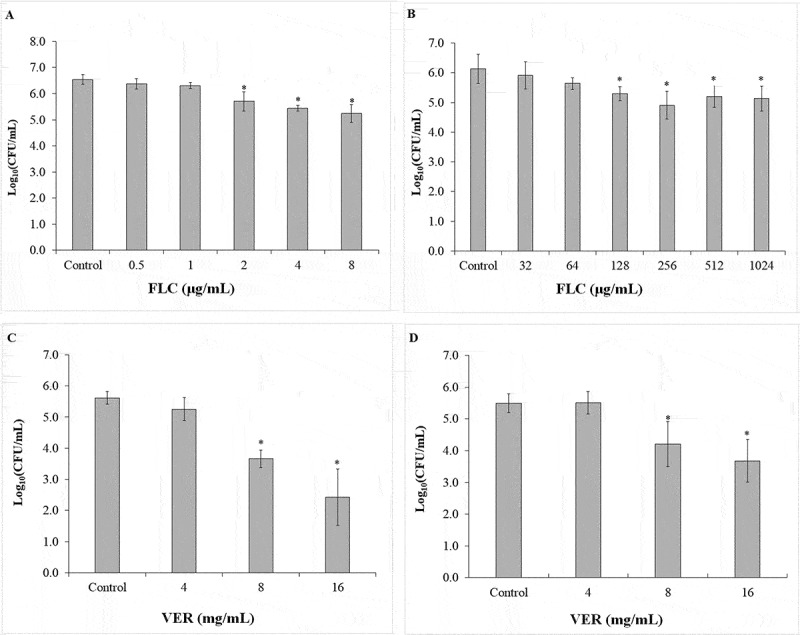
Mean values of log_10_ (CFU/mL) obtained for the biofilms of CaS and CaR strains incubated with FLC and VER for 24 h. FLC for CaS (A) and CaR (B), VER for CaS (C) and CaR (D). Error bars: standard deviation (n = 5). (*) indicates the significant difference between the treated and control groups (*p* < 0.05)

The use of VER and FLC alone did not eradicate biofilm growth. The CFU/mL values obtained for FLC and VER for biofilms in both CaS and CaR are shown in Figure 5. Significant (*p* ≤ 0.001) reductions in CFU/mL were observed after incubation with FLC at concentrations of ≥ 2 μg/mL and ≥ 128 μg/mL for CaS (0.84 to 1.30 log_10_) and CaR (0.84 to 1.23 log_10_), respectively, compared with the respective controls (without drug; Figure 5a and b). VER used at a concentration of 8 and 16 mg/mL promoted significant (*p* ≤ 0.001) reductions of 1.96 and 3.19 log_10_ (CFU/mL), respectively, for CaS biofilms (Figure 5c), and of 1.29 and 1.81 log_10_ (CFU/mL), respectively, for CaR biofilms (Figure 5d) compared with the respective controls.

#### Inhibition of fungal efflux systems

After identification of the drug concentrations that caused a growth reduction of the fungal biofilms, VER was combined with FLC at non-lethal concentrations. A concentration of 4 mg/mL VER was used for both strains of *C. albicans* with 1 and 64 μg/mL FLC used for CaS and CaR, respectively.

**Figure 6.**
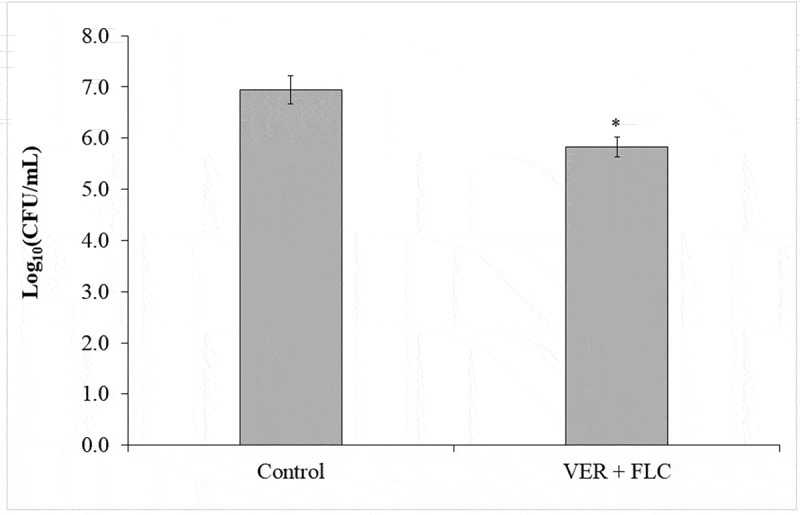
Mean values of log_10_ (CFU/mL) obtained for biofilms incubated for 24 h with 4 mg/mL VER and 1 μg/mL or 64 μg/mL of FLC for CaS and CaR, respectively. Error bars: standard deviation (n = 3). (*) indicates a significance difference between the treated and control groups (*p* < 0.05)

No significant interaction effect (*p* = 0.716) was observed between the strain (CaS, CaR) and the treatment with VER with FLC; however, a significant effect was observed for the strain (*p* = 0.043) and the treatment (*p* < 0.001). A significant (*p* < 0.001) growth reduction (1.11 log_10_) was observed for biofilms treated with a combination of VER and FLC (Figure 6) compared with the control-treated biofilms (without drug). No significant difference (*p* = 0.332) was observed between CaS [6.29 ± 0.60 log_10_ (CFU/mL)] and CaR [6.47 ± 0.68 log_10_ (CFU/mL)].

### In vivo *assays*

#### *Effect of FLC and VER on the survival of* G. mellonella *infected with* C. albicans

The results from *in vivo* assays for *G. mellonella* infected with *C. albicans* showed a reduction in larval survival (*p* ≤ 0.010) in the CaS and CaR strains relative to the control saline-injected groups.

**Figure 7.**
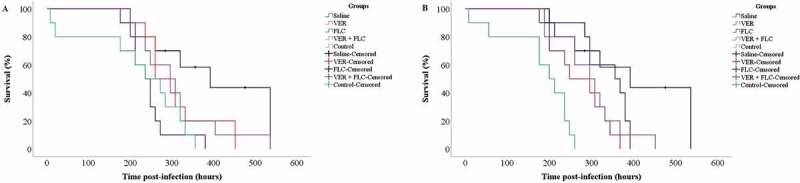
Survival curves for *G. mellonella* infected with the CaS (a) and CaR (b) strains upon treatment with VER and FLC. The groups evaluated were: Saline (sterile saline alone); Control (fungal inoculum and saline); FLC (fungal inoculum and 0.5 and 128 μg/mL of FLC for the CaS and CaR strains, respectively); VER (fungal inoculum and 4 mg/mL VER); VER + FLC (fungal inoculum and 4 mg/mL VER combined with 0.5 and 128 μg/mL of FLC for the CaS and CaR strains, respectively). Censored observations are indicated with a plus sign (+) (data collection was stopped when the larvae became pupae)

The survival analysis for *G. mellonella* infected with CaS (Figure 7a) indicated that the control group did not show a significant difference (*p* ≥ 0.235) in survival outcomes relative to the drug-treated larvae infected with CaS, and the larval groups treated with VER and VER + FLC did not show a significant difference (*p* ≥ 0.071) relative to the saline group.

For larvae infected with CaR, the control group showed the shortest survival times (*p* ≤ 0.012), and all treatments (VER, FLC, VER + FLC) increased the survival time of larvae infected with CaR; however, the difference for the FLC-treated group was not significant (*p* = 0.137) relative to the saline-treated group (Figure 7b).

#### Fungal recovery from G. mellonella

**Figure 8.**
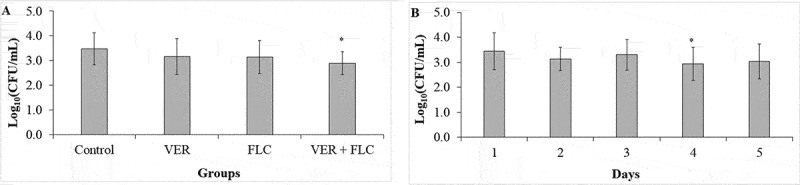
Mean values of log_10_ (CFU/mL) strain concentrations recovered 5 days post-injection from *G. mellonella* infected with CaS or CaR and treated with VER, FLC, or a combination of both drugs. **A**: The difference among the treatment groups regardless of the strain and the recovery day (n = 40); **B**: The difference among the recovery days regardless of the strain and the treatment groups (n = 32). (*)indicates a significant difference (p < 0.05) compared with the control (A) or the day 1 after infection (B). The groups evaluated were: Saline (sterile saline alone; null values for both strains); Control (fungal inoculum and saline); FLC (fungal inoculum and 0.5 and 128 μg/mL of FLC for the CaS and CaR strains, respectively); VER (fungal inoculum and 4 mg/mL VER); VER + FLC (fungal inoculum and 4 mg/mL VER combined with 0.5 and 128 μg/mL of FLC for the CaS and CaR strains, respectively). Error bars: standard deviation

After 5 days of infection with the *C. albicans* strains, the fungal load from the larvae that were treated or untreated with VER and FLC was determined by the recovery of CaS and CaR. The saline-treated group did not result in the recovery of *C. albicans* from the larvae. A three-way ANOVA did not show a significant interaction among the factors (*p* ≥ 0.091); however, each factor when considered alone (strain, treatment, and recovery day) demonstrated a significant effect (*p* ≤ 0.004). The recovery of the CaR strain [3.41 ± 0.70 log_10_ (CFU/mL)] was greater (*p* < 0.001) than that of the CaS strain [2.94 ± 0.53 log_10_ (CFU/mL)]. The use of the combination of VER and FLC significantly reduced (*p* < 0.001) the fungal recovery by 0.58 log_10_ compared with the control (Figure 8a). The fungal recovery was significantly (*p* = 0.016) lower on the fourth day compared with the first day after infection (Figure 8b).

## Discussion

To analyze antifungal resistance *in vivo*, we investigated the inhibition of the efflux system that acts as the main mechanism underlying the resistance toward FLC in *C. albicans* [13–18]. VER has been used as an EPI *in vitro* in *C. albicans* [24–26] and may serve as an important strategy for combating antifungal resistance. Before conducting the *in vivo* assays, we investigated the MIC of all drugs *in vitro*, the inhibition of the efflux system, and the interaction between VER and FLC.

VER showed the same MIC (4 mg/mL) for both strains and higher concentrations (8 and 16 mg/mL) reduced the biofilm viability for CaS and CaR. Another study also reported a reduction in the metabolic activity for biofilm formation and pre-formed biofilm in *C. albicans* treated with VER at concentrations ranging from 40 to 1280 μg/mL [24]. These results may be explained by the inhibitory effect of VER on the virulence of *C. albicans*. The use of VER at concentrations ranging from 20 to 640 μg/mL inhibited *C. albicans* filamentation, adherence to polystyrene surfaces and buccal epithelial cells, expression of the *HWP1* (hyphal wall protein 1) gene, and the gastrointestinal colonization of mice [26]. Moreover, the use of 80 μg/mL VER increased the susceptibility of *C. albicans* toward oxidative stress by reducing the fungal oxidative stress response [25].

Further, we demonstrated that the combination of VER with FLC reversed the resistance of *C. albicans* toward FLC, as a sub-MIC of FLC (64 μg/mL) promoted significant reductions in the log_10_ (CFU/mL) when combined with sub-MIC of VER (2 and 4 mg/mL for planktonic cells and biofilms, respectively). The analysis of the effect of VER and FLC on biofilm formation in *C. albicans* showed that the MIC_50_ of VER was reduced from 160 mg/L to 20 mg/L [24]. On pre-formed biofilms, the MIC_50_ of VER and FLC was reduced from 320 to 80 mg/L and from > 256 to 0.5 mg/L, respectively [24]. In fungal cells growing as part of biofilms in the presence of VER, the use of concentrations ranging from 160 to 1280 mg/L reduced the metabolic activity of biofilms in more than 60% [24]. In contrast, our results showed that only a higher concentration of VER (8 and 16 mg/mL) reduced biofilm viability. This difference may be attributed to the method used to evaluate the effect of the drugs, as in this study we used the quantification of colonies instead of the cellular metabolic activity.

An FLC-susceptible strain was also evaluated in this study as a control. However, the combination of VER with FLC had a stronger effect on the FLC-resistant strain than that observed in the FLC-susceptible one. This result was expected since the susceptible strain does not overexpress the efflux systems, which are the substrate for the inhibitors. However, as a limitation of our investigation, we did not evaluate the expression of *CDR1, CDR2*, and *MDR1* genes to determine the exact mechanism of resistance of *C. albicans*. This evaluation could explain better our susceptibility results and determine if VER is specific to the ABC and/or MFS EP families.

The results of accumulation/efflux assays showed that Rh123 was retained in the CaS, but not in CaR strains, indicating that Rh123 is a substrate for the EPs. We observed that VER increased the intracellular accumulation of Rh123 in CaR, and this may result due to a decrease in the efflux pump activity. A previous study has shown that the accumulation of Rh123 in planktonic *C. albicans* was higher during the earlier than the later phases of growth; therefore, the mid-log phase of growth was used to standardize the assay [41]. However, the authors of the study also reported that 10 μM VER did not increase the accumulation of Rh123 in FLC-resistant *C. albicans*, probably due to shorter exposure and the lower concentration [41]. Another investigation showed higher accumulation of Rh123 in the early phase (6 h) biofilms than in the intermediate (12 h) and mature (48 h) biofilms and planktonic cultures of *C. albicans*, which indicated that the azole resistance of *C. albicans* biofilms mediated by the EPs occurs at the early stage of biofilm growth alone [6].

The combination of VER with FLC at the sub-MIC level showed a synergism in the reduction of the CaR viability, which indicates that VER reversed the resistance toward FLC. Despite not evaluating VER, another study reported synergism of other calcium channel blockers (amlodipine, nifedipine, benidipine, and flunarizine) with FLC against *C. albicans* by the Bliss independence analysis [42]. Altogether, our *in vitro* results demonstrated that VER was an effective EPI and increased the susceptibility of the CaR strain to FLC.

Our *in vivo* results demonstrated that treatment with both VER and FLC increased the larval survival and reduced the fungal recovery for CaR but not for the CaS strains. This result suggests that the combinatorial use of drugs was effective in treating the infection caused by CaR. This result is in accordance with the *in vitro* results that demonstrated a greater reduction in growth for CaR than for the CaS strains. Another study showed that proton pump inhibitors (omeprazole, lansoprazole, pantoprazole, rabeprazole, esomeprazole, and ilaprazole) inhibited the efflux pump activity of FLC-resistant *C. albicans* [43]. The combination of these inhibitors with FLC increased the survival of larvae and reduced the black lumps with yeast and hyphae that were observed in histological sections [43]. The combination of licofelone (dual microsomal prostaglandin E2 synthase/lipoxygenase inhibitor) and FLC also increased the survival of *G. mellonella* infected with an FLC-resistant *C. albicans*, and decreased the fungal burden in the CFU and histological sections, although no effect on the efflux pump was observed [40]. In these studies, larvae treated with the combined drugs showed the greatest survival, whereas in our investigation no difference was observed among groups treated with the drugs alone or together. This result from our investigation may be explained by the MIC used for each drug; we used the MIC as the fungal concentration was increased to more than 1 × 10^7^ CFU/mL to reduce the survival of the larvae (see Supplemental Material).

Another limitation of our study is that we used only one reference strain of FLC-susceptible and -resistant *C. albicans*. Clinical isolates were not evaluated, which may lead to different outcomes owing to distinct virulence activities.

In conclusion, our results from experiments performed *in vitro* showed that VER reverted the FLC-resistance of *C. albicans* and showed synergism with FLC against CaR. The drug increased the survival of *G. mellonella* infected with CaR and reduced the fungal recovery. These results can pave the way for future *in vivo* studies and clinical trials aimed to combat the antifungal resistance using VER as an EPI to reverse FLC resistance. Because VER is an approved drug for clinical use, repurposing its use may shorten the path to the clinical treatment of resistant infections.

## Abbreviation

CaR: fluconazole-resistant *C. albicans*; CaS: fluconazole-susceptible *C. albicans*; CFU: colony forming unit; CI: confidence interval; DMSO: dimethylsulfoxide; EP: efflux pump; EPI: efflux pump inhibitor; FICI: fractional inhibitory concentration index; FLC: fluconazole; MIC: minimum inhibitory concentration; MFC: minimum fungicidal concentration; VER: verapamil; PBS: phosphate-buffered saline; Rh123: Rhodamine 123; subMIC: concentrations lower than the MIC

## Supplementary Material

Supplemental Material
