# Supplementary material for: Verapamil inhibits efflux pumps in Candida albicans, exhibits synergism with fluconazole, and increases survival of Galleria mellonella
Source: Virulence. 2021 Jan 7;12(1):231–43. doi: 10.1080/21505594.2020.1868814 (PMC8923067; doi:10.1080/21505594.2020.1868814)
Supplement: Supplemental Material [file KVIR_A_1868814_SM5896.zip › SUPPLEMENT/Supplemental_materialclean.docx]

**SUPPLEMENTAL MATERIAL**

Studies have shown that certain substances can be used to inhibit EPs in the fungal plasma membrane, such as curcumin (CUR) [1]. When used as an EPI, 100 μM CUR showed selectivity for the ABC transporters of *C. albicans* [2]. In another study, CUR acted synergistically with FLC and showed favorable results for the inhibition of EPs, possibly of the MFS class [1].

Alternative methods have been investigated to overcome drug resistance, such as the use of antimicrobial Photodynamic Therapy (aPDT) [3-6], which associates a photosensitizer (PS) with a light source of appropriate wavelength. The interaction between PS and light in the presence of oxygen results in the production of a toxic reactive species, especially singlet oxygen, which is responsible for cell damage and death [5,7]. However, some PSs, such as phenothiazinium dyes, are substrates of the efflux systems, which protect the cell from photoinactivation [8-10]. According to Prates *et al*., 2011 [9], the overexpression of the efflux systems in *C. albicans*, mainly of the ABC class, abrogated the fungicidal effect of that is aPDT mediated by methylene blue. The inhibition of the efflux system becomes important to allow or even enhance the antimicrobial effect of aPDT [9].

Natural compounds, such as CUR, have shown efficacy in the photoinactivation of *C. albicans* [6,11-14] and resistant bacteria, such as methicillin-resistant *Staphylococcus aureus* [6,15]. CUR is a phenolic compound (diferuloylmethane) found in the rhizomes of *Curcuma longa* L, which has therapeutic properties such as anti-inflammatory, antioxidative, anticancer, and antimicrobial activities [16]. However, to the best of our knowledge, the CUR-mediated photoinactivation of azole-resistant *C. albicans* is unknown.

Therefore, we used CUR (Sigma Aldrich, St. Louis, MO, USA, purity ≥ 65%) as an inhibitor of efflux systems and photosensitizers (PS). The CUR solution was prepared in dimethyl sulfoxide (DMSO, Diadema, SP, Brazil) and was diluted in sterile ultrapure water (DMSO final concentration of 2.5%, Figure S1)


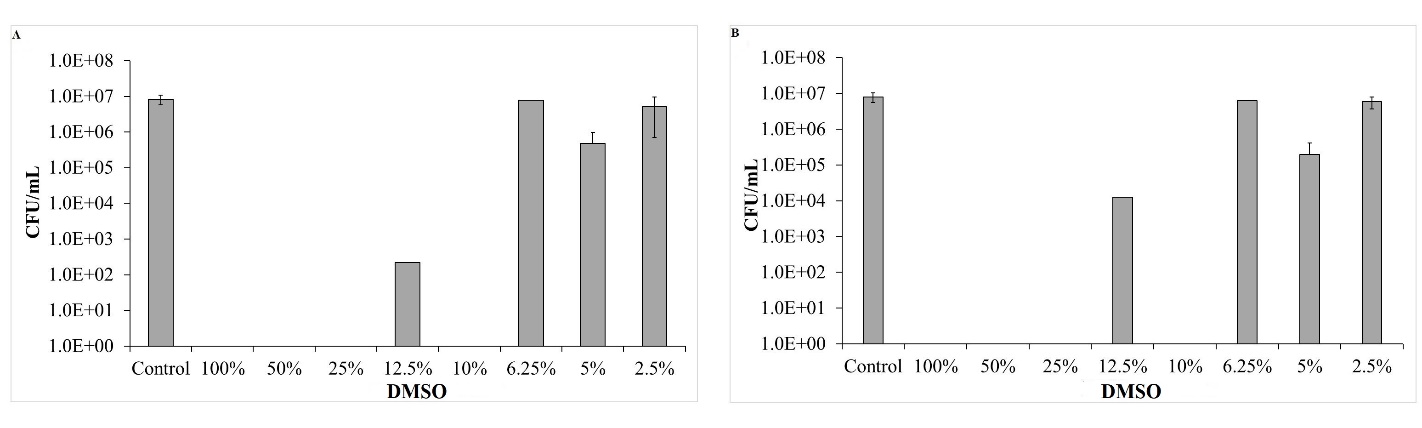


**Figure S1.** Mean values of colony-forming units per milliliter (CFU/mL) obtained for FLC-susceptible *C. albicans* **(A)** and FLC-resistant *C. albicans* **(B)** incubated with different concentrations of DMSO for 24 h (n = 5).

## Light Source for aPDT

A light-emitting diode (LED) device, composed of eight LEDs (LXHL-PR09, Luxeon® III Emitter, Lumileds Lighting, San Jose, CA, USA) and with an average light intensity of 33.58 mW/cm^2^, was used. This device had a uniform emission from 440 to 460 nm (blue spectrum), with maximum emission at ≈455 nm, and was developed by the São Carlos Institute of Physics – University of São Paulo (USP).

## Planktonic Cultures

**Susceptibility Test**

The MIC and MFC of CUR were evaluated as described in section 2.3.1 (manuscript). The final CUR concentrations used ranged from 0.625 to 40 μM for CaS and from 40 to 5120 μM for CaR.

**Inhibition of Fungal Efflux Systems**

Non-lethal concentrations (sub-MIC) of CUR (10 μM for CaS and 40 μM for CaR) were combined with FLC (0.25 μg/mL for CaS and 64 μg/mL for CaR) for each strain as described in the section 2.3.2 (manuscript). Moreover, the combination of the three drugs (VER, CUR, and FLC) was also evaluated.

**Interaction of EPIs with FLC**

The interaction of CUR with FLC was evaluated following the same methodology described in section 2.3.4 (manuscript). The final concentrations of CUR and FLC used for each strain are shown in Table S1.

**Table S1.** Concentrations of drugs used in the Checkerboard assay.

| Drugs | CaS | CaR |
| --- | --- | --- |
| FLC (µg/mL) | 0, 0.032, 0.063, 0.125, 0.25, 0.5 | 0, 0.5, 1, 2, 4, 8, 16, 32, 64, 128 |
| CUR (µM) | 0, 0.63, 1.25, 2.5, 5, 10 | 0, 0.63, 1.25, 2.5, 5, 10, 20, 40 |

CUR: curcumin; FLC: fluconazole; CaS: FLC-susceptible *C. albicans*; CaR: FLC-resistant *C. albicans*.

**Antimicrobial Photodynamic Therapy (aPDT) against Planktonic Cultures**

For the aPDT assay, aliquots of 100 μL of the fungal suspension from each standardized strain (see section 2.2.) were individually transferred to wells from a 96-well, flat-bottom microtiter plate (TPP Techno Plastic Products, Trasadingen, Switzerland). For the aPDT (CUR and light: C+L+ group), the same volume (100 μL) of CUR was pipetted into these wells to obtain a final concentration of 40 μM. The samples were incubated for 5 min (pre-irradiation time, PIT) in the dark for photosensitization, followed by LED illumination at 5.28 J/cm^2^ [11], corresponding to 2.6 min. Control samples were treated only with CUR in the dark for the same PIT and illumination time (7.6 min, C+L- group), only with LED light (5 min, 5.28 J/cm^2^, C-L+ group) after incubation with sterile PBS, or those that received no treatment (PBS only for 7.6 min, C-L- or control group). For the evaluation of fungal viability, the samples were diluted in PBS and plated onto SDA plates, which were incubated at 37˚C for 48 h for colony quantification.

**Biofilms**

## Susceptibility Test

The CUR susceptibility test was performed as described in section 2.4.1 (manuscript). The final CUR concentrations used ranged from 10 to 160 μM for CaS and from 40 to 640 μM for CaR.

**Inhibition of Fungal Efflux Systems**

The highest non-lethal concentration of CUR was combined with the highest non-lethal concentration of FLC. The concentrations of CUR and FLC were 10 μM and 1 μg/mL, respectively, for CaS, and 40 μM and 64 μg/mL, respectively, for CaR. We did not associate either EPIs, VER and CUR, with FLC for biofilms, as we did for planktonic cultures, because the combination VER+CUR+FLC did not cause a reduction in fungal viability in the planktonic assays (see Results section 5.1.3 from the manuscript).

**Photodynamic Treatment Against *C. albicans* Biofilms**

After biofilm formation, the culture medium was carefully aspirated from each well and the samples were washed twice with 200 μL of sterile PBS. Due to the greater tolerance towards biofilms compared to its planktonic counterpart, the concentration of CUR in the biofilm was higher than in planktonic tests. Next, 200 μL of CUR at 80 μM were added to each well and incubated for 20 min (PIT) in the dark, followed by illumination for 20 min, equivalent to 40.3 J/cm^2^ (C+L+ group). The same groups described above for the planktonic cultures were evaluated for biofilm formation (C+L-, C+L+ and C-L-). As aPDT with VER did not increase the photokilling of either strain in planktonic cultures compared with aPDT without VER (see Results), we did not perform the aPDT with VER for biofilms. After treatments, each biofilm sample was washed with PBS and mechanically disrupted using a pipette tip and 200 μL of PBS for serial dilutions, which were plated on SDA and incubated at 37ºC for 48 h for colony counting.

***In vivo* Assays**

In the pilot study, we used fungal suspensions at the concentration of 4.34×10^6^ ± 6.81×10^5^ CFU/mL for larvae inoculation as reported by other studies [17-19]; however, it did not reduce larval survival (Figure S2). This result might be attributed to the different susceptibilities of larvae toward candida infection. Therefore, we employed higher concentrations for larval inoculation (section 3.1 from the manuscript). Regarding the EPI, only VER was associated with FLU for the *in vivo* assays, because the association of VER and FLU resulted in higher fungal killing than the combination of CUR and FLU (see Results section from the manuscript, see section 5.1.3 and Figure S3).


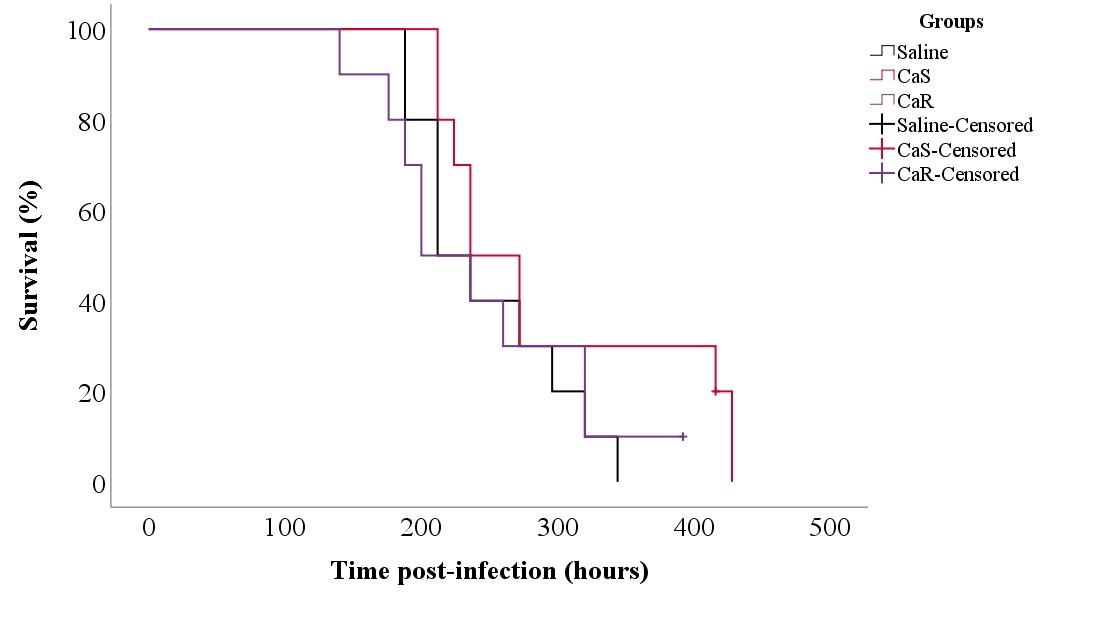


**Figure S2.** Survival curve of *G. mellonella* infected with CaS and CaR. All larvae were infected with 4.34×10^6^ CFU/mL (n = 10). CaS: FLC-susceptible *C. albicans*; CaR: FLC-resistant *C. albicans*; FLC: fluconazole.

***In vivo* Fungal Photoinactivation**

To perform aPDT (C+L+ group), larvae were inoculated with one fungal suspension as previously described (section 3.1 of the manuscript). An aliquot of 10 µL of 40 and 80 μM CUR for CaS and CaR, respectively, was also injected. After 5 min (PIT), the larvae were transferred to wells of a 24-well plate (Kasvi) and were irradiated using a blue LED (455 nm, 33.6 mW/cm^2^) for 2.6 min (5.28 J/cm^2^). The other groups (C+L-, C-L+, and C-L-) were also evaluated, and larvae from the saline group received saline and the CUR vehicle (2.5% DMSO). After the injections, the larvae were kept in separate Petri dishes according to each group (n = 10), incubated at 33ºC, and observed daily for survival until no larvae remained.

**Fungal Recovery from *G. mellonella***

The fungal load was determined as described in the section 3.2 (manuscript). The larvae were divided into the same groups (n = 10) as described above (*in vivo* fungal photoinactivation).

**Results and Discussion**

**Susceptibility Test**

Our results showed that the MIC of CUR was identified only for CaS (20 μM or 7.37 μg/mL, reduction of 1.40 log_10_), but not for CaR. This result did not agree with those from a previous study, which observed the fungistatic effect of CUR, with MIC values ranging from 5 to 20 mM against clinical isolates and laboratory strains of *C. albicans* that were susceptible and resistant to FLC [20]. Evaluating a reference strain of *C. albicans* (ATCC 18804), Martins *et al*. [16] identified MIC values of 64 and 2 mg/L for CUR and FLC, respectively. Another investigation reported MIC values ranging from 250 to 2000 μg/mL for CUR and from 8 to > 64 μg/mL for FLC against planktonic cultures of the reference strains and clinical isolates of *C. albicans* [21]. These studies identified higher MIC values for CUR than that observed in our investigation, which may be attributed to the strains used, purity and source of CUR, and methodological variations. The anticandidal mechanism of CUR has been reported to be caused via cell wall damage [22] and apoptosis caused by reactive oxygen species [23].

**Inhibition of the Fungal Efflux Systems**

CUR (10 μM) was combined with FLC (0.25 μg/mL) for testing in the CaS strain. Although the MIC of CUR was identified for CaR, 40 μM CUR combined with 64 μg/mL FLC was used for the CaR strain. Previously, 40 μM CUR alone was tested against the CaR strain and the results showed a mean ± SD of 6.65 ± 0.55 log_10_ (CFU/mL) (n = 4), a value similar to the control of 6.60 ± 0.06 log_10_ (CFU/mL). For the combination of CUR with FLC, there was a significant interaction (*p* < 0.001) between the strain and the treatment. CUR with FLC resulted in a higher reduction of CaR (2.28 log_10_, *p* < 0.001) than for CaS (1.65 log_10_, *p* < 0.001; Figure S3A).

When the three drugs were combined, a two-way ANOVA indicated that a significant interaction (*p* < 0.001) existed between the strain and the treatment. When both EPIs (CUR and VER) were combined with FLC, a significant reduction in the CFU/mL was observed. After incubation, there were reductions in the colony growth of 0.83 log_10_ (*p* < 0.001) and 2.86 log_10_ (*p* < 0.001) for the CaS and CaR strains (Figure S3B), respectively, compared with their controls (without drugs).

The combination of CUR and FLC increased the susceptibility of CaR towards FLC, and a similar result was observed by Garcia *et al*.; 2012 [1] who found that 11 μM CUR showed synergism with 4 mg/L FLC, reducing in ~80% growth of a clinical isolate of *C. albicans* that was resistant towards FLC (MIC: 256 mg/L). When used as EPIs, 100 μM CUR was selective for ABC transporters of *C. albicans*, acting as a competitive inhibitor of the efflux system and showed synergism with ketoconazole, itraconazole, and miconazole but not with FLC [2]. This result does not corroborate that observed in this study, and we found that CUR increased the susceptibility of resistant *C. albicans* towards FLC. This difference may be explained by the strains used in both the investigations. Our study used an FLC-resistant *C. albicans* strain, whereas Sharma *et al.* 2009 [2] evaluated a strain of *Saccharomyces cerevisiae* overexpressing the EPs of *C. albicans* in their assays. Moreover, they used mutant strains that overexpressed the specific efflux transporters (*Cdr1p* and *Cdr2p* from ABC transporters and *Mdr1p* from MFS transporters) [2]. As a limitation of our investigation, the expression of the *CDR1*, *CDR2*, and *MDR1* genes was not evaluated to determine the exact mechanism of resistance in *C. albicans*. Therefore, CUR may have increased the fungal susceptibility towards FLC by other mechanisms other than efflux pump inhibition.


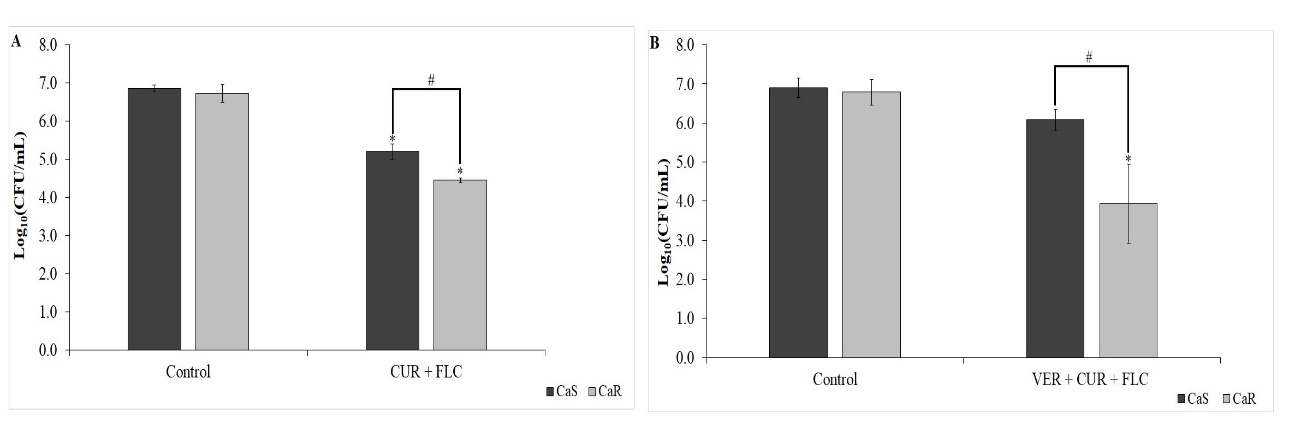


**Figure S3.** Mean values of log_10_ (CFU/mL) identified for both strains after 24 h of incubation with: **(A)** 10 μM (CaS) or 40 μM CUR (CaR) with 0.25 μg/mL FLC (CaS) or 64 μg/mL FLC (CaR); **(B)** VER at 2 mg/mL, 10 μM (CaS) or 40 μM (CaR) CUR, and 0.25 μg/mL FLC (CaS) or 64 μg/mL (CaR) were used. Error bars: standard deviation (n = 3). (*****) indicates a significance difference existed between the treated and control groups in the same strain and (**#**) indicates a significant difference between the strains in the same treatment (*p* < 0.05). FLC: fluconazole; CUR: curcumin; CaS: FLC-susceptible *C. albicans*; CaR: FLC-resistant *C. albicans*.

**Interaction of EPIs with FLC**

The checkerboard assay using CUR with FLC for CaS showed synergism (FICI value of 0.0915) between 0.63 μM CUR with 0.03 μg/mL FLC. However, the plated samples showed high CFU/mL mean values. For CaR, FICI was not calculated, since no MIC of CUR was identified for this strain, and the plated samples showed mean values ranging from 8.20×10^3^ to 8.81×10^6^ CFU/mL.

The Bliss independence analysis for CaS demonstrated synergism between 1.25 µM CUR combined with 0.063 and 0.032 µg/mL FLC [Figure S4A, confidence intervals (CI) of 0.4252 – 0.1864 and 0.5021 – 0.1643] and antagonism between 5 µM CUR with 0.032 µg/mL FLC (CI of -0.0041 - -0.2893) and 2.5 µM CUR with 0.125 µg/mL FLC (CI of -0.0256 - -0.7467). For CaR (Figure S4B) synergism was observed when 40 µM CUR was combined with 64, 32, and 16 µg/mL FLC (CI of 5.8895 – 1.6359, 4.0285 – 1.8768, and 2.8671 – 0.2593, respectively), 64 µg/mL FLC with 20 µM CUR (CI of 4.2880 – 0.0817) and 32 µg/mL FLC with 5 µM CUR (CI of 0.6902 – 0.1034).

The combination of subMIC values of CUR with FLC showed synergism for CaR, which demonstrated that CUR reversed the resistance to FLC. Our findings agree with those of Garcia-Gomes *et al*. [1], who also observed synergism of CUR with FLC against *C. albicans* strains, but disagree with those of Sharma *et al*. [2], who did not observe synergism of CUR with FLC against *Saccharomyces cerevisiae* that overexpressed the ABC and MFS transporters (*Cdr1p*, *Cdr2p*, and *Mdr1p*). In another study, Sharma *et al*. [24] reported synergism between CUR and antifungal agents (polyenes and azoles, including fluconazole) against wild-type and clinical isolates of *C. albicans*. Synergism between CUR and FLC was also observed against planktonic growth and biofilm formation in a reference strain of *C. albicans* that showed resistance to FLC (ATCC 10231). However, the abovementioned studies evaluated the interaction of the drugs only by the FICI method and not using a Bliss independence analysis.


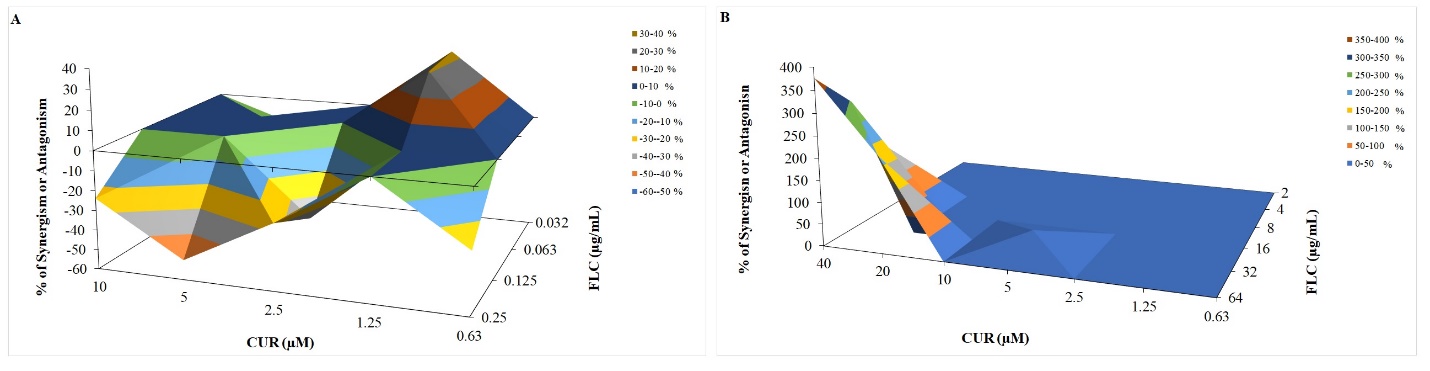


**Figure S4.** Three-dimensional surface graph representing the Bliss independence analysis of the interaction between CUR and FLC for CaS **(A)** and CaR **(B)**. The concentrations of CUR and FLC are shown in X and Z-axes, and the Y-axis shows the %ΔE. Peaks above the 0 plane represent synergism (%), valleys below the 0 plane represent antagonism (%), and the 0 plane represents no interaction (95% CI overlapped with 0). FLC: fluconazole; CUR: curcumin; CaS: FLC-susceptible *C. albicans*; CaR: FLC-resistant *C. albicans*.

**Photodynamic Treatment Against Planktonic Cultures**

For aPDT (Figure S5), there was no significant (*p* = 0.103) interaction between the strain and the treatment, and a significant effect (*p* < 0.001) was found only for the treatment. The 40 μM CUR treated (C+L-) group alone promoted a significant reduction (*p* = 0.021) of 2.34 log_10_ of CaS. On the other hand, no reduction was observed for CaR (*p* = 0.977), when compared with their respective controls (C-L-). When CUR was combined with light (5.28 J/cm^2^, C+L+ group), both strains showed a significant reduction (*p* < 0.001) of 4.50 and 4.42 log_10_, respectively, in comparison with their respective controls. The treatment with LED light alone (C-L+ group) did not cause a significant difference (*p* = 0.331) compared with the control**.** We also evaluated aPDT in the presence of VER. aPDT with 40 μM CUR and 2 mg/mL VER resulted in a mean ± SD of 3.03 ± 1.20 and 3.28 ± 0.71 log_10_ (CFU/mL) for the CaS and CaR strains, respectively (n = 4).

In the aPDT assay, 40 μM CUR alone reduced the viability of CaS by 2.34 log_10_ of planktonic, whereas in the MIC assay the same concentration reduced the fungal viability by 6.06 log_10_. This difference in the reduction values can be explained by the incubation time of CaS with CUR, as in the aPDT assay this period was 5 min, whereas in the MIC assay this period was 24 h. The aPDT mediated by 40 μM CUR in combination with LED light at 5.28 J/cm^2^ promoted a significant reduction in the viability of the two *C. albicans* strains (4.50 and 4.42 log_10_ for CaS and CaR, respectively). Therefore, the susceptibility of CaR towards aPDT was similar to that of CaS, which is an important finding in the current scenario of the fight against microbial resistance. These results differ from previous data reported by Dovigo *et al*. [11], who identified the complete inactivation of planktonic *C. albicans* that were susceptible to FLC when 20 μM CUR was combined with LED fluence of 5.28 J/cm^2^ after 5 and 20 min of PIT. Another study [25] demonstrated that aPDT mediated by 20 μM CUR in combination with LED light (5.28 J/cm^2^) resulted in the complete inactivation of planktonic *C. albicans*, and the fungal cells treated with CUR alone did not show a reduction in viability compared to their respective controls. These differences in the results can be attributed to the methodological differences between the studies; while these authors evaluated an overnight fungal inoculum prepared with the Trypticase Soy Broth culture medium and CUR was prepared in 10% DMSO, in our study the inoculum was prepared in the mid-log phase in YNB culture medium and CUR was prepared with 2.5% DMSO. Our results agree in part with those of a previous study in which higher parameters of aPDT (130 μM CUR and 43.2 J/cm^2^ LED) eradicated the colony growth of *C. albicans* and treatment with CUR alone reduced 2.43 log_10_ of the fungal viability [6]. Although we evaluated aPDT with 2 mg/mL VER only on one occasion, the reduction observed was lower than that verified for aPDT without VER. A previous study also demonstrated that 50 μM VER reduced the photokilling of *C. albicans* by methylene blue and laser light [26], suggesting that the transport of calcium is important for photoinactivation.


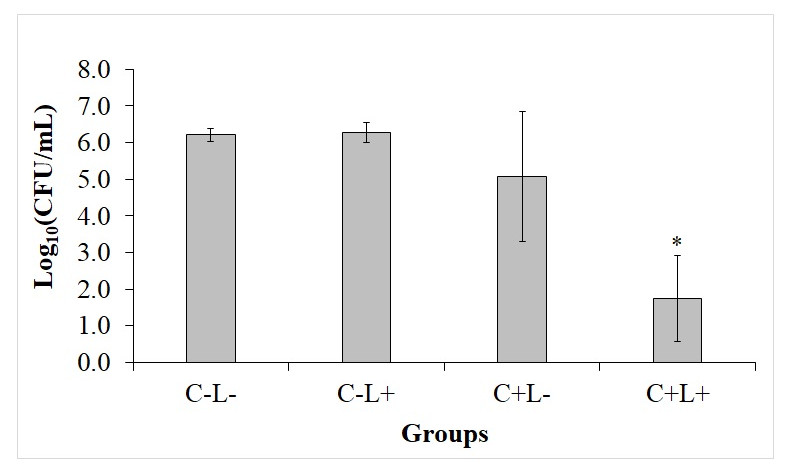


**Figure S5.** Average and standard deviation (error bars, n = 3) values of log_10_ (CFU/mL) obtained for the CaS and CaR strains. C-L-: control group; C-L+: fungal suspensions treated only with light (455 nm, 5.28 J/cm^2^); C+L-: fungal suspensions treated only with 40 μM CUR; C+L+: fungal suspensions treated with aPDT (CUR and LED light). (*****) indicates significance difference between treated and control groups (*p* < 0.05). CUR: curcumin; CaS: FLC-susceptible *C. albicans*; CaR: FLC-resistant *C. albicans*.

**Biofilms**

**Susceptibility Test**

CUR did not eradicate biofilm growth. A reduction in the CFU/mL values for biofilms was not observed for either strain after incubation with CUR at all concentrations analyzed, compared with the drug vehicle (*p* ≥ 0.100; Figure S6).

For biofilms, CUR did not reduce the viability of the CaS and CaR strains, although the MIC value of CUR (20 μM or 7.37 μg/mL) reduced the colony growth of planktonic CaS alone by 1.40 log_10_. This result did not corroborate those found in other studies, which observed a reduction in *C. albicans* biofilms using higher concentrations of CUR. Shahzad *et al*. [27] demonstrated that 50 μg/mL CUR reduced the metabolic activity of sessile *C. albicans* and the biofilm biomass when CUR was added at the adhesion phase of biofilm formation. CUR used at concentrations of 30 and 60 mM inhibited biofilm formation by 60% and the mature biofilm by 38%, respectively, in an FLC-resistant strain of *C. albicans* (ATCC 10231) [20]. Another study [28] showed that the MIC values of CUR were 100 and 200 μg/mL for planktonic cultures and biofilms, respectively, for a laboratory strain of *C. albicans* (SC5314). The difference between these results and that from our study may be ascribed to the strains used, the biofilm age, and the methodology employed to assess the biofilms, as most studies evaluated the effect of CUR on biofilm formation after 24 h by analyzing the metabolic activity. It was shown that a reduction of metabolic activity may not correspond to a reduction of viability in colony growth [29], as cells may recover from a sublethal stress, which reduces its metabolic activity; however, the cells are still able to grow as a colony during the incubation period. Therefore, the method of colony quantification (CFU) has been recommended to evaluate the antimicrobial effect of new methods and drugs [30,31].


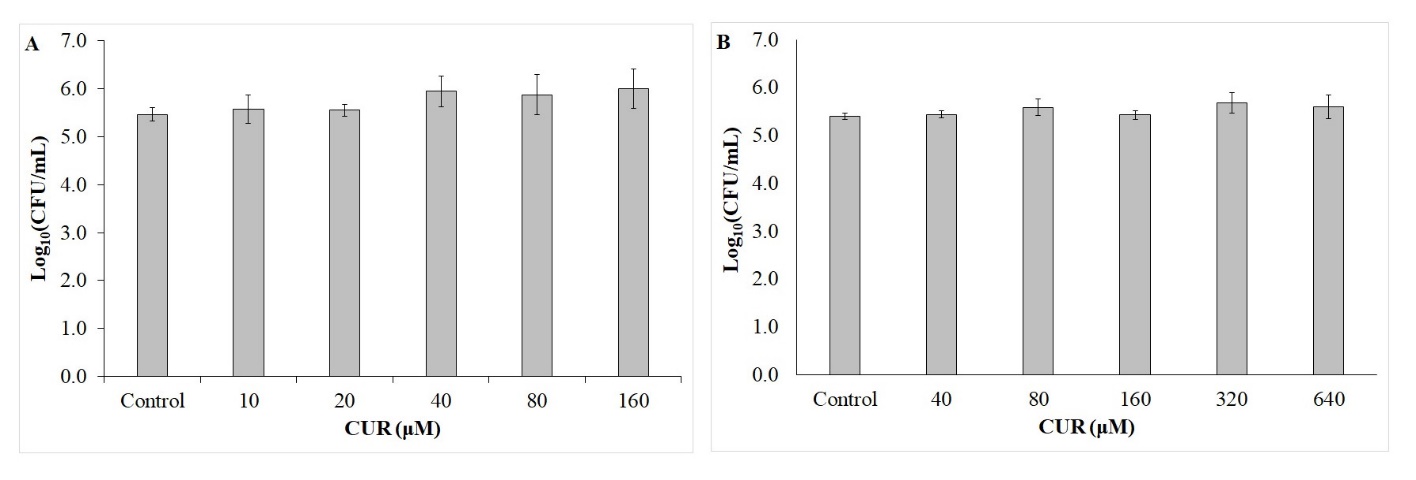


**Figure S6**. Mean values of log_10_ (CFU/mL) were obtained for biofilms of CaS **(A)** and CaR **(B)** that were incubated with CUR for 24 h. Error bars: standard deviation (n = 5). CUR: curcumin; CaS: FLC-susceptible *C. albicans*; CaR: FLC-resistant *C. albicans*.

**Inhibition of Fungal Efflux Systems**

CUR was used at concentrations of 10 and 40 μM for the CaS and CaR strains, respectively, combined with 1 and 64 μg/mL FLC for the CaS and CaR strains, respectively. For biofilms treated with CUR+FLC, no significant interaction between the strain and the treatment (*p* = 0.542) was verified, and neither of the main factors demonstrated a significant effect (*p* ≥ 0.152, mean ± SD of 5.70 ± 0.92 and 5.38 ± 0.21 log_10_ (CFU/mL) for CaS and CaR, respectively, and 6.03 ± 0.53 and 6.16 ± 0.57 log_10_(CFU/mL) for controls CaS and CaR, respectively).

**Photoinactivation of *C. albicans***

For aPDT, there was no significant (*p* = 0.598) interaction between the strain and the treatment, and a significant effect (*p* < 0.001) was found for the treatment alone. When 80 μM CUR was combined with light (40.3 J/cm^2^, C+L+ group), biofilms showed a significant reduction of 1.78 log_10_ (*p* < 0.001) compared with the control. CUR (C+L- group) at 80 μM did not promote a significant reduction (*p* = 0.199) in biofilm viability when compared with the control (C-L-) group. The treatment with LED light alone (C-L+ group) did not indicate a significant difference (*p* = 0.863) relative to the control (Figure S7)**.**

The aPDT was also effective for reducing the viability of biofilms, which agrees with the results of other studies that used CUR and LED light. CUR at 60 μM with LED at 7.92 J/cm^2^ reduced the metabolic activity of *C. albicans* biofilms from 66.44% to 90.87% [32]. Reductions in the metabolic activity of *C. albicans* biofilms by up to 95% were observed after aPDT mediated by 5 to 40 μM CUR [11,25]. The combination of 40 μM CUR and 18 J/cm^2^ LED also reduced the total biomass of the *C. albicans* biofilms [12]. A higher concentration of CUR at 1200 μM, combined with a higher light fluence at 43.3 J/cm^2^ reduced the *C. albicans* biofilm by 1.24 log_10_ [6]. Nonetheless, these investigations demonstrated that planktonic cells are more susceptible to CUR-mediated aPDT than the biofilms of *C. albicans*. Therefore, new strategies should be further developed to increase the susceptibility of biofilms to aPDT.


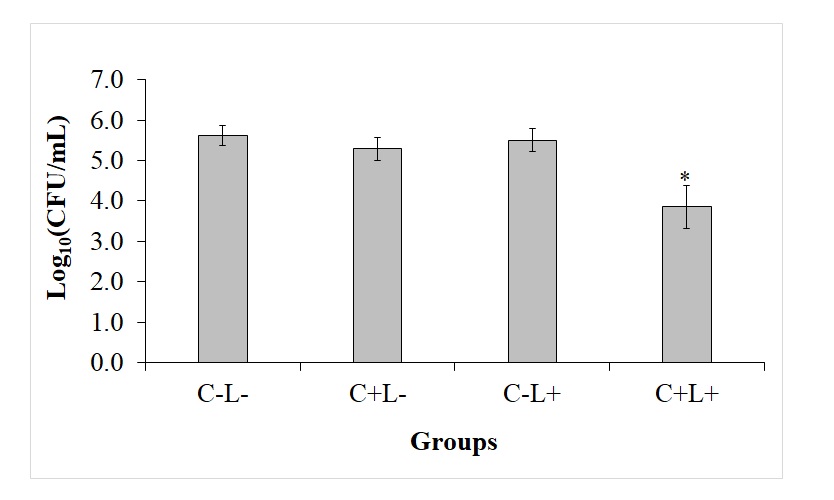


**Figure S7.** Mean values of log_10_ (CFU/mL) obtained for biofilms analyzed for aPDT mediated by 80 μM CUR and LED light (455 nm) at 40.3 J/cm^2^. Error bars: standard deviation (n = 3). C-L-: control group; C+L-: fungal suspensions treated with 80 μM CUR alone; C-L+: fungal suspensions treated with light alone (455 nm, 40.3 J/cm^2^); C+L+: fungal suspensions analyzed via aPDT (CUR and LED light). (*****) indicates a significant difference between the treated and control groups. CUR: curcumin.

***In vivo* Fungal Photoinactivation**

For the survival curve of *G. mellonella* infected with CaS (Figure S8A), the larvae analyzed via aPDT (C+L+ group) did not show a significant difference (*p* = 0.407) with the saline group (non-infected control), and both groups showed the longest survival. The other groups (C-L-, C+L-, and C-L+) showed a significantly (*p* ≤ 0.001) shorter survival time compared to the aPDT and saline groups.

In the survival curve of *G. mellonella* infected with CaR (Figure S8B), the saline group alone showed the longest survival (*p* ≤ 0.001) compared with the other groups (C-L-, C+L-, C-L+, and C+L+), which did not show significant differences (*p* > 0.290) among themselves. Therefore, for CaR, the aPDT did not increase the larvae survival times.


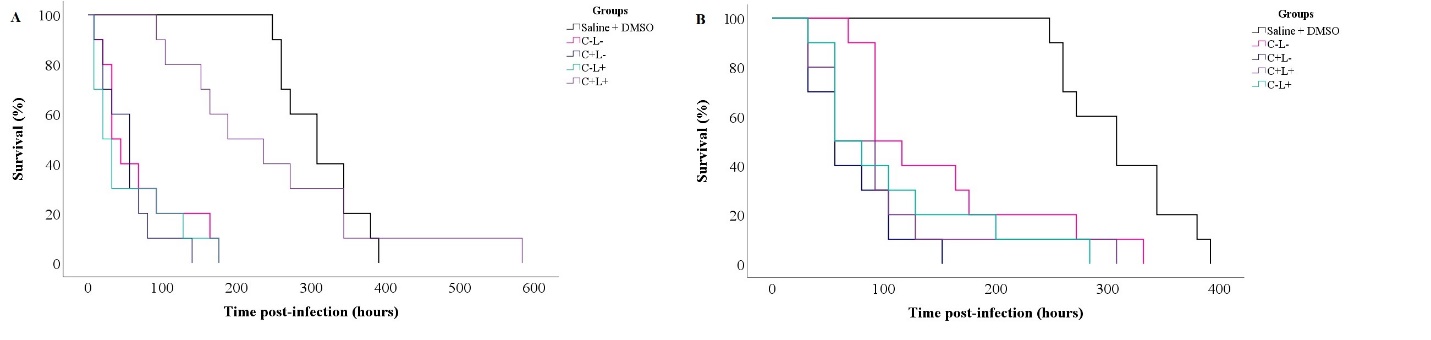


**Figure S8.** Survival curve of *G. mellonella* infected with CaS **(A)** and CaR **(B)** analyzed via aPDT. The evaluated groups were: C-L- (fungal inoculum and saline + DMSO, control); C+L+ (fungal inoculum and 40 and 80 μM CUR for CaS and CaR, respectively, combined with light at 5.28 J/cm^2^, aPDT); C-L+ (fungal inoculum and LED light at 5.28 J/cm^2^); C+L- (fungal inoculum and 40 and 80 μM CUR for CaS and CaR, respectively); Saline + DMSO (saline and 2.5% DMSO)**.** CUR: curcumin.

**Fungal Recovery from *G. mellonella***

For the *in vivo* photoinactivation assay, we did not observe a significant interaction (*p* ≥ 0.503) among the factors nor any significant effects (*p* ≥ 0.644) for any of the factors. Therefore, the fungal recovery was similar between the strains and among the recovery days and the treatment groups (Figure S9).


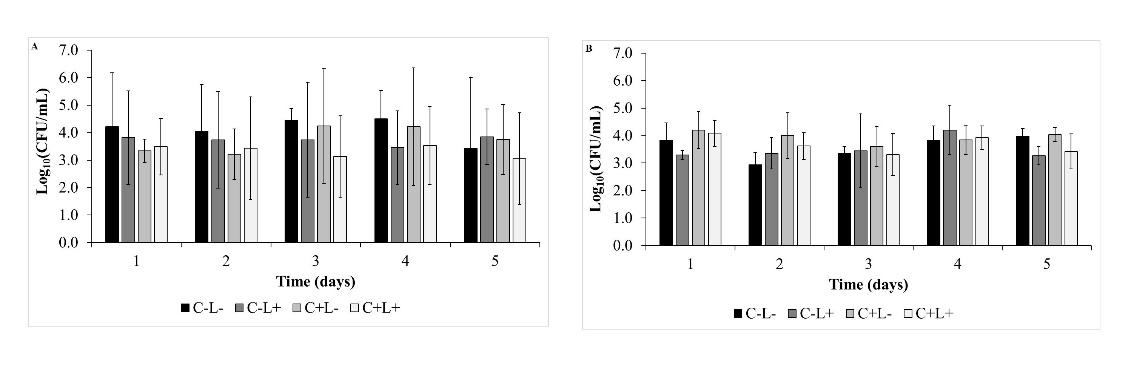


**Figure S9.** Mean values of log_10_ (CFU/mL) after 5 days of *G. mellonella* infected with CaS **(A)** and CaR **(B)** and analyzed via aPDT mediated by 40 and 80 μM CUR for CaS and CaR, respectively, and LED irradiation (455 nm, 5.28 J/cm^2^). The evaluated groups were: C-L- (fungal inoculum and 2.5% DMSO, control); C+L+ (fungal inoculum and CUR + light, aPDT); C-L+ (fungal inoculum and LED light); C+L- (fungal inoculum and CUR); Saline (only sterile saline, null values for both strains). Error bars: standard deviation (n = 4). CUR: curcumin.

In the *in vivo* photoinactivation assay, the aPDT increased the survival for larvae infected with CaS alone, although no significant difference was observed in the CFU/mL recovered from larvae among the groups. This unexpected result observed for the CFU/mL of CaS may be explained by the high standard deviation observed in this assay (Figure S10A), as the mean CFU/mL of the aPDT group (3.33 ± 1.36) was lower than that of the control group (4.13 ± 2.27). In contrast to the *in vitro* assays, we did not observe *in vivo* photoinactivation of CaR, as demonstrated by the larval survival times and CaR recovery. This outcome may be explained by the higher concentration of fungal inoculum used in larvae than that used in the *in vitro* assays, as the higher the microbial density, the lower the photoinactivation observed [33]. Another study verified that CUR and light increased the survival of the larvae infected with *C. albicans* [34]. In agreement with our results, Chibebe Junior *et al*. [35] demonstrated that aPDT mediated by methylene blue also increased larval survival in those infected with an FLC-susceptible *C. albicans* alone, but not with an FLC-resistant strain. However, they observed a reduction in the CFU/mL recovered from the larvae that were analyzed via aPDT [35]. The use of CUR as a PS for aPDT may be advantageous over methylene blue, as the latter is a substrate for EPs, leading to reduced photoinactivation [9].

**Supplementary References**

[1] Garcia-Gomes AS, Curvelo JAR, Soares RMA, Ferreira-Pereira A. Curcumin acts synergistically with fluconazole to sensitize a clinical isolate of *Candida albicans* showing a MDR phenotype. Med. Mycol. 2012; 50: 26-32. doi:10.3109/13693786. 2011.578156.

[2] Sharma M, Manoharlal R, Shukla S, Puri N, Prasad T, Ambudkar S, *et al*. Curcumin modulates efflux mediated by yeast ABC multidrug transporters and is synergistic with antifungals. Antimicrob. Agents Chemother. 2009; 53: 3256-3265. doi:10.1128/AAC.01497-08.

[3] Pereira Gonzales F, Maisch T. Photodynamic inactivation of microorganisms as an innovative approach to kill mucocutaneous and skin microorganisms. G. Ital. Dermatol. Venereol. 2010; 145: 477-489.

[4] Soukos NS, Goodson JM. Photodynamic therapy in the control of oral biofilms. Periodontol 2000. 2011; 55: 143-166. doi:10.1111/j.1600-0757.2010.00346.x.

[5] Donnelly RF, McCarron PA, Tunney MM. Antifungal photodynamic therapy. Microbiol. Res. 2008; 163: 1-12. doi:10.1016/j.micres.2007.08.001.

[6] Trigo Gutierrez JK, Zanatta GC, Ortega ALM, Balastegui MIC, Sanitá PV, Pavarina AC, *et al*. Encapsulation of curcumin in polymeric nanoparticles for antimicrobial photodynamic therapy. PLoS One. 2017; 12: e0187418. doi:10.1371/journal.pone.0187418.

[7] Jori G, Fabris C, Soncin M, Ferro S, Coppellotti O, Dei D, *et al*. Photodynamic therapy in the treatment of microbial infections: basic principles and perspective applications*.* Lasers Surg. Med. 2006; 38: 468-481. doi:10.1002/lsm.20361.

[8] Kishen A, Upadya M, Tegos GP, Hamblin MR. Efflux pump inhibitor potentiates antimicrobial photodynamic inactivation of *Enterococcus faecalis* biofilm. Photochem. Photobiol. 2010; 86: 1343-1349. doi:10.1111/j.1751-1097.2010.00792.x.

[9] Prates RA, Kato IT, Ribeiro MS, Tegos GP, Hamblin MR. Influence of multidrug efflux systems on methylene blue-mediated photodynamic inactivation of *Candida albicans*. J. Antimicrob. Chemother. 2011; 66: 1525-1532. doi:10.1093/jac/dkr160.

[10] Tegos GP, Hamblin MR. Phenothiazinium antimicrobial photosensitizers are substrates of bacterial multidrug resistance pumps. Antimicrob. Agents Chemother. 2006; 50: 196-203. doi: 10.1128/AAC.50.1.196-203.2006.

[11] Dovigo LN, Pavarina AC, Ribeiro AP, Brunetti IL, Costa CA, Jacomassi DP, *et al*. Investigation of the photodynamic effects of curcumin against *Candida albicans*. Photochem. Photobiol. 2011; 87: 895-903. doi:10.1111/j.1751-1097.2011.00937.x.

[12] Dovigo LN, Pavarina AC, Carmello JC, Machado AL, Brunetti IL, Bagnato VS. Susceptibility of clinical isolates of *Candida* to photodynamic effects of curcumin. Lasers Surg. Med. 2011; 43: 927-934. doi:10.1002/lsm.21110.

[13] Dovigo LN, Carmello JC, de Souza Costa CA, Vergani CE, Brunetti IL, Bagnato VS, et al. Curcumin-mediated photodynamic inactivation of Candida albicans in a murine model of oral candidiasis. Med. Mycol. 2013; 51: 243-251. doi:10.3109/13693786.2012.714081.

[14] Sakima VT, Barbugli PA, Cerri PS, Chorilli M, Carmello JC, Pavarina AC, et al. Antimicrobial photodynamic therapy mediated by curcumin-loaded polymeric nanoparticles in a murine model of oral candidiasis. Molecules. 2018; 23: 2075. doi:10.3390/molecules23082075.

[15] Liu J, Yu M, Zeng G, Cao J, Wang Y, Ding T, et al. Dual antibacterial behavior of a curcumin-upconversion photodynamic nanosystem for efficient eradication of drug-resistant bacteria in a deep joint infection. J. Mater. Chem. B. 2018; 6: 7854-7861. doi:10.1039/c8tb02493f.

[16] Martins CV, Da Silva DL, Neres AT, Magalhães TF, Watanabe GA, Modolo LV, et al. Curcumin as a promising antifungal of clinical interest. J. Antimicrob. Chemother. 2009; 63: 337-339. doi:10.1093/jac/dkn488.

[17] Fuchs BB, O'Brien E, Khoury JB, Mylonakis E. Methods for using *Galleria mellonella* as a model host to study fungal pathogenesis. Virulence. 2010; 1: 475-482. doi:10.4161/viru.1.6.12985.

[18] Liu X, Li T, Wang D, Yang Y, Sun W, Liu J, *et al*. Synergistic antifungal effect of fluconazole combined with licofelone against resistant *Candida albicans*. Front. Microbiol. 2017; 8: 2101. doi:10.3389/fmicb.2017.02101.

[19] Brennan M, Thomas DY, Whiteway M, Kavanagh K. Correlation between virulence of *Candida albicans* mutants in mice and *Galleria mellonella* larvae. FEMS Immunol. Med. Microbiol. 2002; 34: 153-157. doi:10.1111/j.1574-695X.2002.tb00617.x.

[20] Thakre AD, Mulange SV, Kodgire SS, Zore GB, Karuppayil SM. Effects of cinnamaldehyde, ocimene, camphene, curcumin and farnesene on *Candida albicans*. Adv. Microbiol. 2016; 06: 627-643. doi:10.4236/aim.2016.69062.

[21] Neelofar K, Shreaz S, Rimple B, Muralidhar S, Nikhat M, Khan LA. Curcumin as a promising anticandidal of clinical interest. Can J. Microbiol. 2011; 57: 204-210. doi:10.1139/W10-117.

[22] Kumar A, Dhamgaye S, Maurya IK, Singh A, Sharma M, Prasad R. Curcumin targets cell wall integrity via calcineurin-mediated signaling in *Candida albicans*. Antimicrob. Agents Chemother. 2014; 58: 167-175. doi:10.1128/AAC.01385-13.

[23] Sharma M, Manoharlal R, Puri N, Prasad R. Antifungal curcumin induces reactive oxygen species and triggers an early apoptosis but prevents hyphae development by targeting the global repressor TUP1 in *Candida albicans*. Biosci. Rep. 2010; 30: 391-404. doi:10.1042/BSR20090151.

[24] Sharma M, Manoharlal R, Negi AS, Prasad R. Synergistic anticandidal activity of pure polyphenol curcumin I in combination with azoles and polyenes generates reactive oxygen species leading to apoptosis. FEMS Yeast Res. 2010; 10: 570-578. doi:10.1111/j.1567-1364.2010.00637.x.

[25] Andrade MC, Carvalho M, Ribeiro D, Dovigo LN, Brunetti I, Giampaolo ET, *et al*. Effect of different pre-irradiation times on curcumin-mediated photodynamic therapy against planktonic cultures and biofilms of *Candida* spp. Arch. Oral Biol. 2013; 58: 200-210. doi:10.1016/j.archoralbio.2012.10.011.

[26] Carvalho GG, Felipe MP, Costa MS. The photodynamic effect of methylene blue and toluidine blue on *Candida albicans* is dependent on medium conditions. J. Microbiol. 2009; 47: 619-623. doi:10.1007/s12275-009-0059-0.

[27] Shahzad M, Sherry L, Rajendran R, Edwards CA, Combet E, Ramage G. Utilising polyphenols for the clinical management of *Candida albicans* biofilms. Int. J. Antimicrob. Agents. 2014; 44: 269-273. doi:10.1016/j.ijantimicag.2014.05.017.

[28] Alalwan H, Rajendran R, Lappin DF, Combet E, Shahzad M, Robertson D, *et al*. The anti-adhesive effect of curcumin on *Candida albicans* biofilms on denture materials. Front. Microbiol. 2017; 8: 659. doi:10.3389/fmicb.2017.00659.

[29] Quishida CC, Carmello JC, Mima EG, Bagnato VS, Machado AL, Pavarina AC. Susceptibility of multispecies biofilm to photodynamic therapy using Photodithazine®. Lasers Med. Sci. 2015; 30: 685-694. doi:10.1007/s10103-013-1397-z.

[30] Nakonieczna J. Comment on "Effectiveness of antimicrobial photodynamic therapy (AmPDT) on *Staphylococcus aureus* using phenothiazinecompound with red laser". Lasers Med. Sci. 2017; 32: 1667-1668. doi:10.1007/s10103-016-2107-4.

[31] Cieplik F, Tabenski L, Buchalla W, Maisch T. Antimicrobial photodynamic therapy for inactivation of biofilms formed by oral key pathogens. Front. Microbiol. 2014; 5: 405. doi:10.3389/fmicb.2014.00405.

[32] Ma J, Shi H, Sun H, Li J, Bai Y. Antifungal effect of photodynamic therapy mediated by curcumin on *Candida albicans* biofilms in vitro. Photodiagn. Photodyn. Ther. 2019; 27: 280-287. doi:10.1016/j.pdpdt.2019.06.015.

[33] Demidova TN, Hamblin MR. Effect of cell-photosensitizer binding and cell density on microbial photoinactivation. Antimicrob. Agents Chemother. 2005; 49: 2329-2335. doi:10.1128/AAC.49.6.2329-2335.2005.

[34] Merigo E, Conti S, Ciociola T, Fornaini C, Polonelli L, Lagori G, *et al*. Effect of different wavelengths and dyes on *Candida albicans*: *In vivo* study using *Galleria mellonella* as an experimental model. Photodiagn. Photodyn. Ther. 2017; 18: 34-38. doi:10.1016/j.pdpdt.2017.01.181.

[35] Chibebe JJ, Sabino CP, Tan X, Junqueira JC, Wang Y, Fuchs BB, *et al*. Selective photoinactivation of *Candida albicans* in the non-vertebrate host infection model *Galleria mellonella*. BMC Microbiol. 2013; 13: 217. doi:10.1186/1471-2180-13-217.
