# Supplementary figures and images for: Verapamil inhibits efflux pumps in Candida albicans, exhibits synergism with fluconazole, and increases survival of Galleria mellonella
Source: Virulence. 2021 Jan 7;12(1):231–43. doi: 10.1080/21505594.2020.1868814 (PMC8923067; doi:10.1080/21505594.2020.1868814)

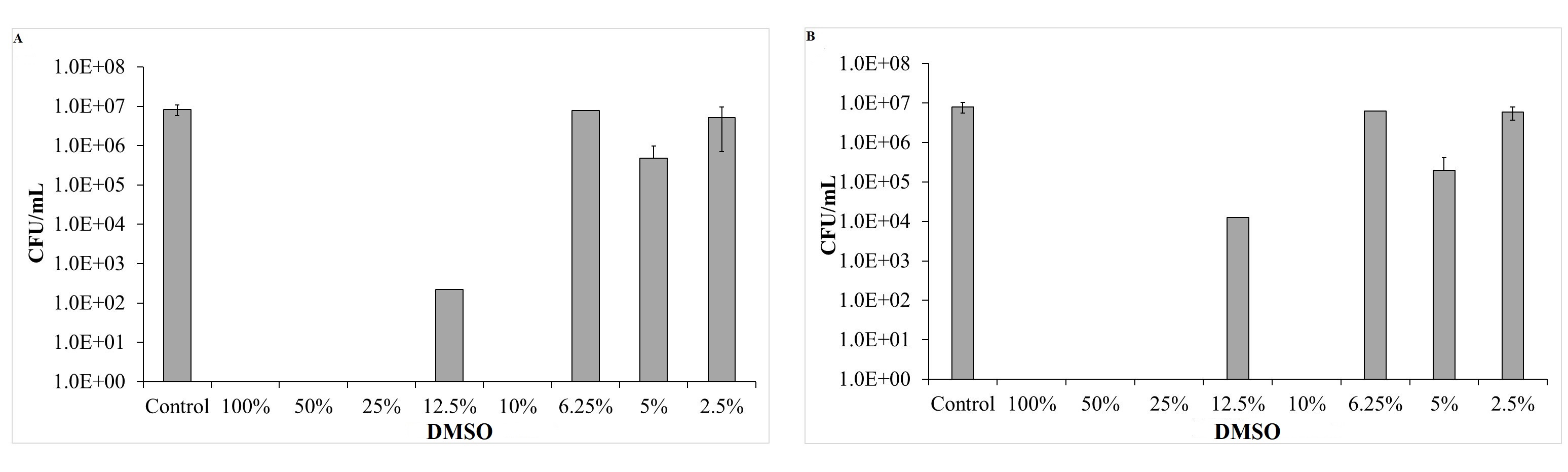

Supplement: Supplemental Material [file KVIR_A_1868814_SM5896.zip › SUPPLEMENT/Figure S1.jpg]

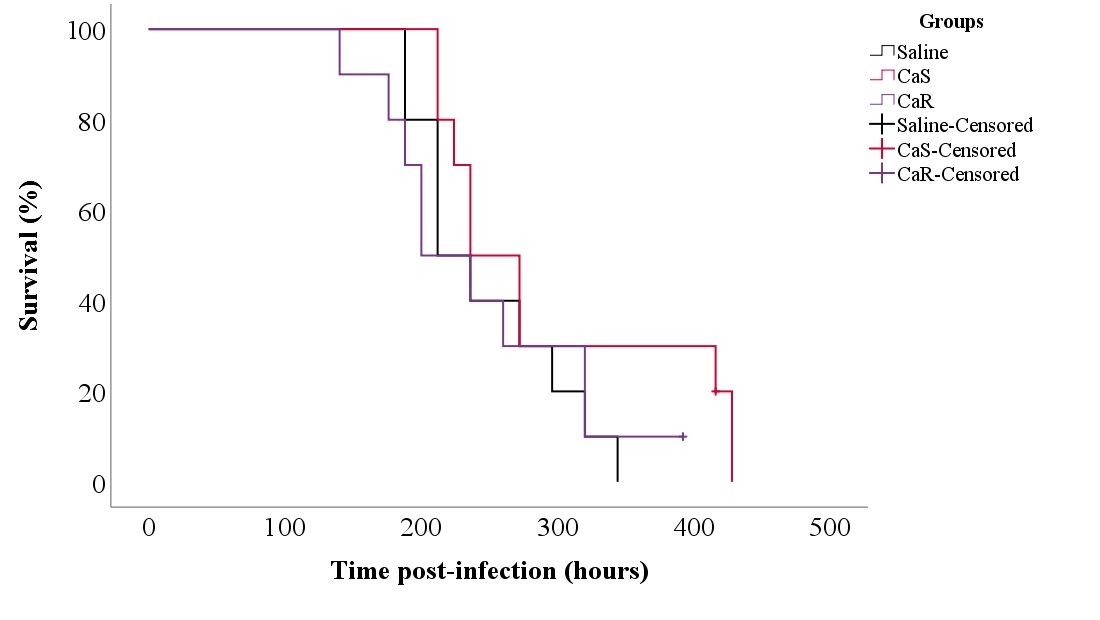

Supplement: Supplemental Material [file KVIR_A_1868814_SM5896.zip › SUPPLEMENT/Figure S2.jpg]

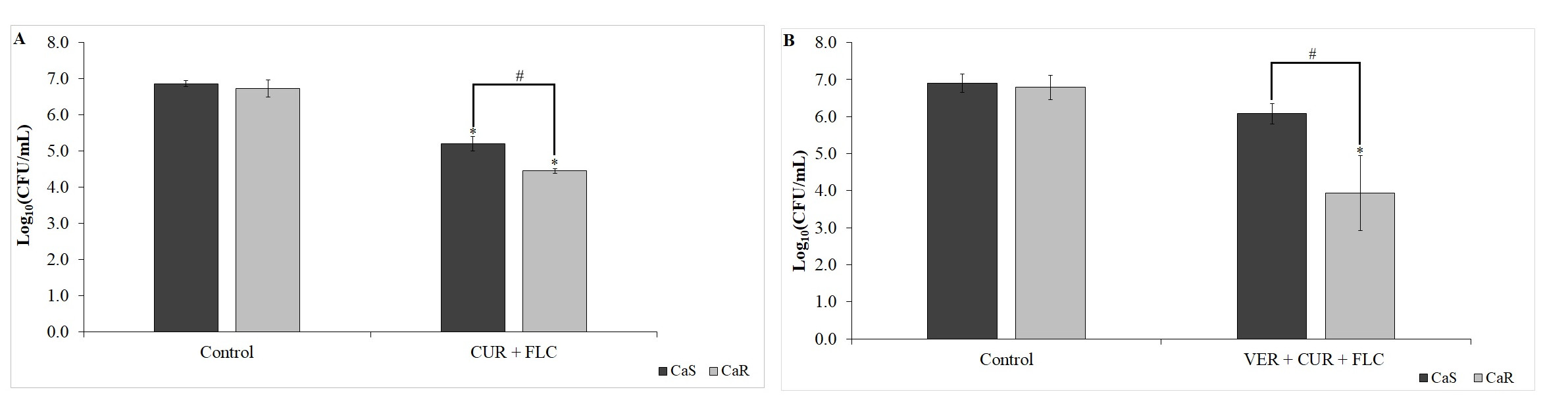

Supplement: Supplemental Material [file KVIR_A_1868814_SM5896.zip › SUPPLEMENT/Figure S3.jpg]

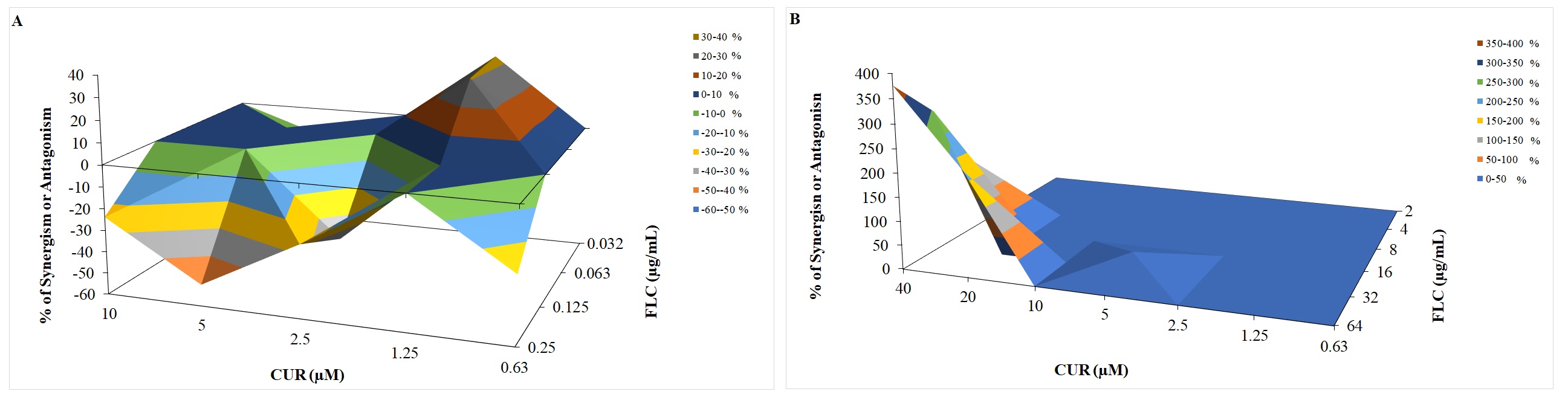

Supplement: Supplemental Material [file KVIR_A_1868814_SM5896.zip › SUPPLEMENT/Figure S4.jpg]

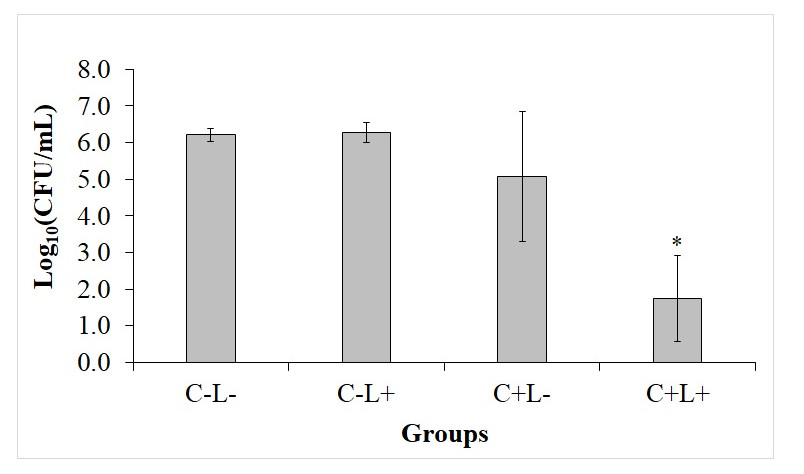

Supplement: Supplemental Material [file KVIR_A_1868814_SM5896.zip › SUPPLEMENT/Figure S5.jpg]

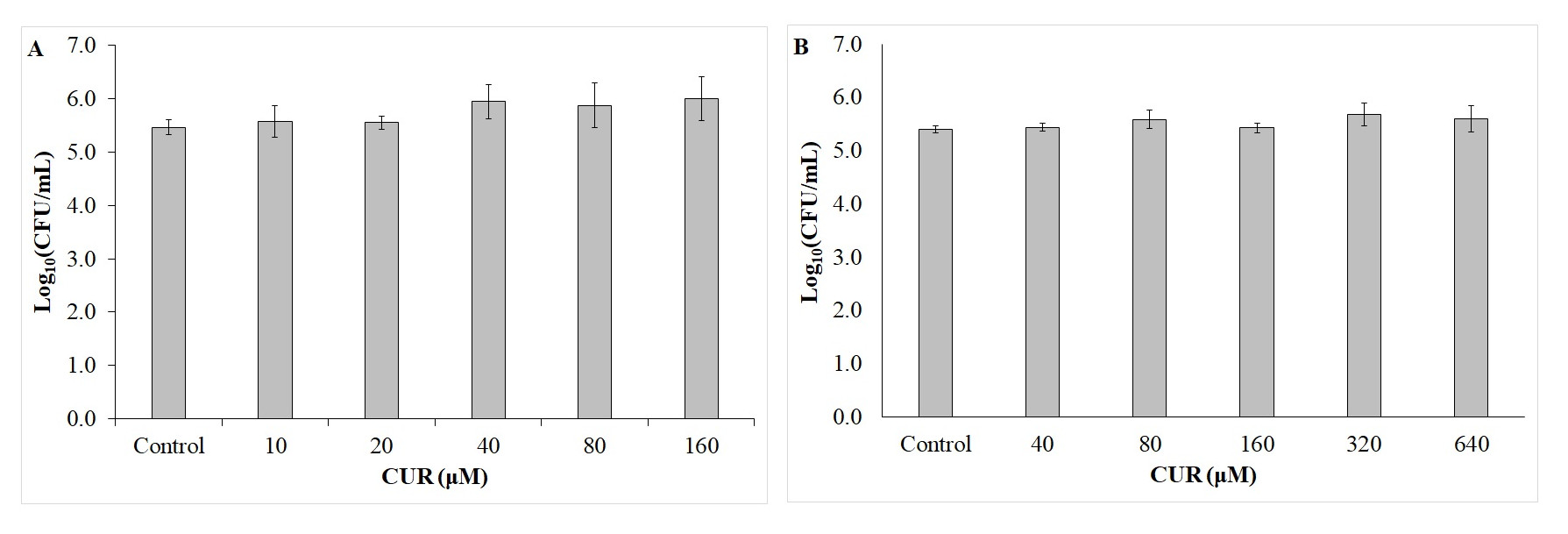

Supplement: Supplemental Material [file KVIR_A_1868814_SM5896.zip › SUPPLEMENT/Figure S6.jpg]

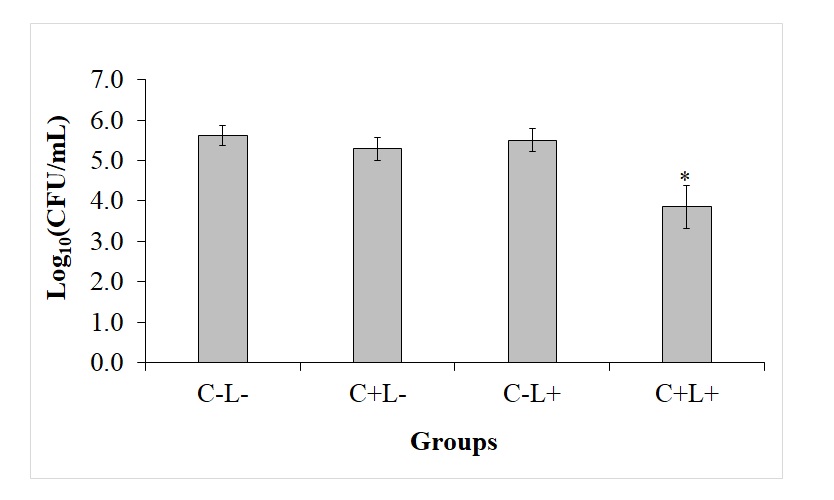

Supplement: Supplemental Material [file KVIR_A_1868814_SM5896.zip › SUPPLEMENT/Figure S7.jpg]

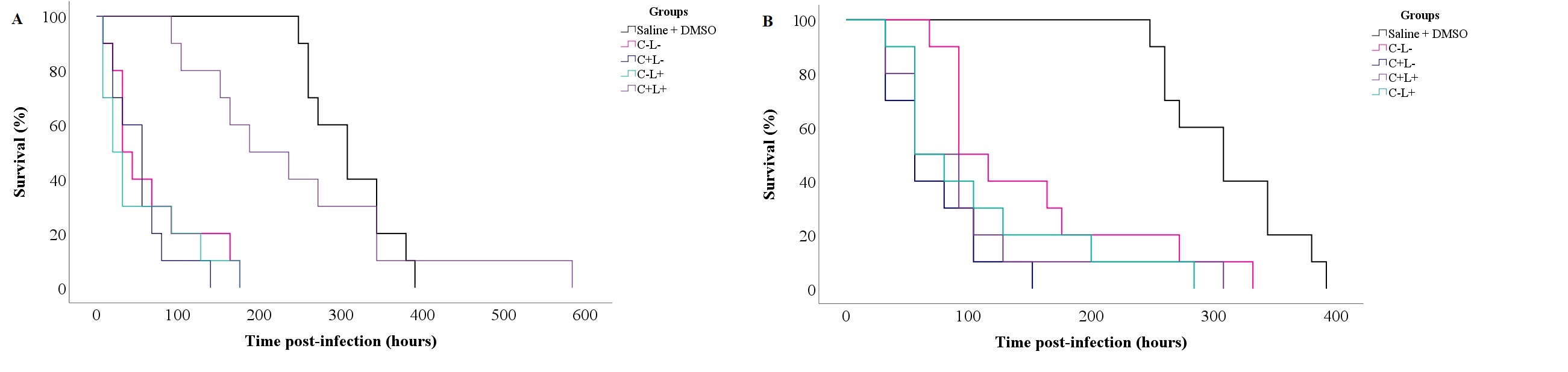

Supplement: Supplemental Material [file KVIR_A_1868814_SM5896.zip › SUPPLEMENT/Figure S8.jpg]

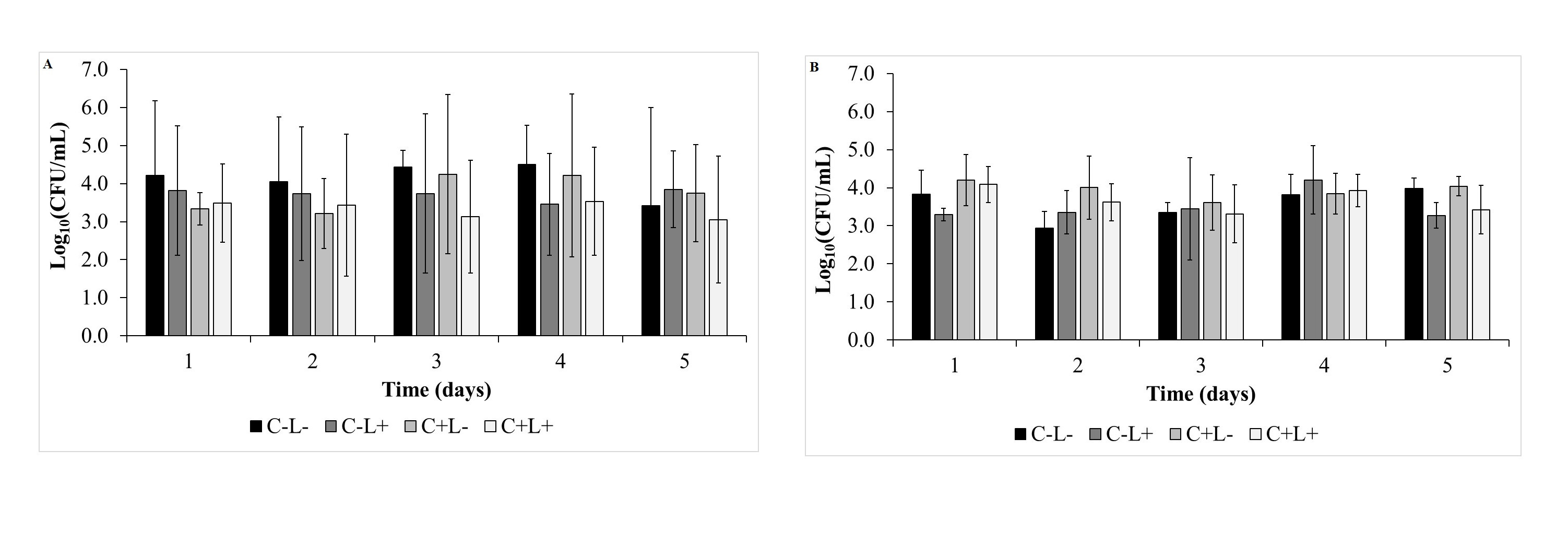

Supplement: Supplemental Material [file KVIR_A_1868814_SM5896.zip › SUPPLEMENT/Figure S9.jpg]
